# Radon gas mapping for environmental assessment in Dessie, Ethiopia

**DOI:** 10.1038/s41598-025-21201-4

**Published:** 2025-10-24

**Authors:** Gebeyaw Endris Ahmed, Hailu Geremew Zeleke

**Affiliations:** 1 Department of Physics, Mekdela Amba University, Tulu Awulia, Ethiopia; 2 Department of Physics, Wollo University, Dessie, Ethiopia

**Keywords:** Radon gas, Emanation coefficient, Geological formation, Geospatial methods, Climatic conditions, Experimental nuclear physics, Theoretical nuclear physics

## Abstract

The geological formations of Dessie town contain rocks that release radon gas, the second leading cause of lung cancer after smoking. To date, there have been no investigations of geology-based radon health risks in Dessie town. The main objective of this study was to evaluate and map the temporal and spatial distribution of radon gas concentrations within Dessie town. The evaluation and mapping were done using advanced geological and geospatial techniques, employing Quantum Geographic Information Systems (QGIS) software to integrate the research area’s weather conditions and geological data using shape files for the study area and masking with its geological map. Geological analysis helps to know whether the geological formation of Dessie town is composed of basaltic rocks, as shown on the geological map of Dessie town, which was obtained from the Ethiopian Geological Institute. Based on these geological formations and geospatial analysis, radon gas activity concentrations were estimated in two different ranges: from 3$\:581.5{\:\text{B}\text{q}\text{m}}^{-3}\:\text{t}\text{o}\:\text{10,744.5}{\:\text{B}\text{q}\text{m}}^{-3}$for the emanation coefficient 0.3 and from $\:\:35.815\:{\:\text{B}\text{q}\text{m}}^{-3}\:\text{t}\text{o}\:107.\:445\:{\text{B}\text{q}\text{m}}^{-3}\:\:$for the emanation coefficient 0.1 for this study area. The radon gas distribution map indicates radon levels in Dessie town fall into two ranges, each with its own potential hotspots, as shown by circles of different colors on the map. These results indicate that there exist health-related problems at nine stations due to prolonged exposure to radon gas, and for further investigation of radon gas activity concentrations and their health impact, we recommend experimental measurements provide more accurate results.

**Supplementary Information:**

The online version contains supplementary material available at 10.1038/s41598-025-21201-4.

## Introduction

Radon (Rn) is a naturally occurring, colorless, odorless, and tasteless radioactive gas. It’s a noble gas, meaning it’s chemically inert, but its radioactivity makes it a significant environmental concern. Since it is a natural part of the environment, its environmental significance primarily stems from its health risks when it accumulates in enclosed spaces and matters environmentally as a major public health hazard, its accumulation in indoor environments, and its environmental journey. It is formed from the natural radioactive decay of uranium and thorium, which are present in varying amounts in nearly all rocks and soils. It is the main contributor to the radiation exposure received by the population from natural background radiation. It exists naturally in the form of three isotopes: ^222^Rn (Radon), ^220^Rn (Thoron), and ^219^Rn (Actinon). Radon-222 is the most stable and environmentally relevant isotope, which is formed by alpha decay of ^226^Ra (Radium), and ultimately from ^238^U^1^. Even though the level of uranium varies from location to location, uranium can be found in trace amounts in all soils and rocks^2^.

The major source of radon in the atmosphere, at least 80% is emanations from soil that is derived from rocks by the mechanical and chemical breakdown. Most of the time, the amount of uranium in the soil will be roughly equal to the amount in the rock^3^. Geological factors are determining factors in the production and distribution of radon, and the presence and concentration of uranium (^238^U) will determine the amount of radon emitted, as highlighted by^4^. On the other hand^5^, show that local geological characteristics, such as rock permeability, soil porosity, geological structure, and topography, directly affect the transport and accumulation of radon.

A study by^6^ in UK shows that radon (^222^Rn) is a particularly important natural environmental hazard over uranium-rich rocks in some parts of the UK. The uranium content of basalt and sedimentary rocks can vary significantly depending on the specific composition and geological history of the rocks. Based on the reports by^7^, the global range of uranium abundance in basalt rock is in the range of 0.1-1 ppm.

A study conducted by^8^ indicates that radon concentration primarily depends on the lithology, structural attributes, and presence of uranium minerals in rocks. According to reports^9^, typical outdoor radon concentration ranges between $\:1\:and\:100\:Bq{m}^{-3},\:$with an estimated annual average of around $\:10\:Bq{m}^{-3}$. According to^10^, outdoor radon could contribute to radon dose, especially in regions where outdoor radon concentrations are higher or close to the indoor ones.

The geospatial distribution of radon gas is not uniform and is primarily influenced by geological factors. Radon, a naturally occurring radioactive gas, is found in trace amounts in all soils and rocks, but its concentration varies significantly based on the presence and abundance of uranium in the underlying geological formations. Local geological characteristics, such as rock permeability, soil porosity, geological structure, and topography, also play a crucial role in the release and movement of radon. Geospatial techniques, particularly GIS and remote sensing, are increasingly utilized in radon mapping to assess and predict radon hazards, aiding in public health and environmental management. According to^11^, a study conducted on the Geographical distribution of radon and associated health risks in drinking water samples collected from the Mulazai area of Peshawar, Pakistan, using Geospatial methods, such as GIS and remote sensing, maps radon levels, pinpoints high-risk areas, and connects geological traits to radon presence. Similarly, another study done in Ghana shows that radon mapping and seasonal radon studies have been carried out within the communities around the Ghana Atomic Energy Commission (GAEC), using the ArcMap geostatistical interpolation tool. Radium concentration was found to have a considerable effect on indoor radon and radon exhalation in dwellings and soils^12^.

Radon transport theory, encompassing concepts like permeability and emanation coefficient modeling, is fundamental to developing effective geospatial strategies for identifying and managing radon risk. It provides the scientific basis for predicting where radon is likely to accumulate and helps in allocating resources for mitigation and public health interventions. Permeability of rocks and soils guides geospatial strategy by identification of high-risk geological formations, soil permeability mapping, and predictive modeling inputs. According to^13^, soils and rocks with higher permeability, such as sandstones and limestones, have larger pores and fissures, allowing radon to move more easily. Emanation coefficient guides geospatial strategy by mapping radium/uranium concentrations, linking to geological units, identification of “radon prone areas” (RPAS) and Calculating Geogenic Radon Potential (GRP) as a study conducted by^14^ soil gas radon and soil permeability assessment: mapping radon risk areas in perak state, malaysia shows that the GRP map assists in human health risk assessment and risk reduction since it indicates the potential of the source of radon and can serve as a vital tool for radon combat planning. By understanding and incorporating permeability and emanation coefficient modeling, geospatial strategies for radon risk assessment become robust and predictive.

Radon concentration levels are strongly affected by atmospheric influences such as rainfall, soil moisture, wind speed, humidity, temperature, and barometric pressure, in addition to geochemical processes, as it is an inert gas^4^. Climatic factors can affect the amount of radon that is released from soil and, in turn, the concentration of radon outside. As noted by^15^, an increase in temperature or wind speed raises the radon flux, but an increase in air pressure, rainfall, and snowfall decreases it.

According to^16^, the movement of radon through faults, fractures, and other openings in the rock can be enhanced by factors such as soil moisture, temperature, and the concentration of other gases in the subsurface. According to a study by^17^, climate factors like temperature, humidity, and precipitation also have an impact on radon transmission. Temperature changes can affect the pressure in the soil, leading to variations in the mobility of radon as reviewed by^18^. Soil moisture can have an impact on radon’s permeability and diffusion capacity^19^. Wet soil can serve as a barrier to radon gas diffusion, reducing radon mobility. Overwhelming rains can saturate the soil, which hinders radon diffusion and increases the possibility that groundwater will carry it^20^. However, because soil moisture is lower in drier areas, radon diffusion may be more effective. A study conducted by^21^ found that when the temperature inside is lower than the outdoors, radon concentrations decrease as the relative humidity rises from 30% to 60%.

According to^22^, increased precipitation can also act to impede radon emanation, “capping” the soil outdoors and directing it toward the unsaturated soil near or under the building. In addition, if the soil is not saturated, low and moderate levels of soil moisture provide a greater radon source that can penetrate through holes in the substructure of a building.

According to^23^, radon and its progeny concentrations showed a diurnal variation pattern with a minimum in the late afternoon and a maximum in the early morning. Similarly, seasonal variation studied by^24^ shows that indoor radon concentrations are highest in winter, during the heating season. As a study performed in Milan, Italy, a seasonal pattern was observed; higher concentrations in winter (around $\:15Bq{m}^{-3}$ ) than in summer (around $\:5Bq{m}^{-3}$ ), by a factor of 3. Higher values in winter were also measured in Turkey by a factor of 2, as highlighted by^25^; a nationwide survey in Japan^26^ and China^27^ by an average factor of 1.8. However, other studies indicate that the lowest values were in winter, presumably due to snow cover, high humidity, or strong winds^28,29^.

A study conducted by^30^ found that the annual average outdoor radon concentration measured for a few consecutive years did not show significant ($\:<15\%$) differences between annual concentrations. Another experiment by^22^ that was performed in 4 consecutive years has shown that variations of annual average radon concentration were up to 15% and up to 30% for 2 locations, respectively. In 4 years of continuous monitoring in Milan, Italy^24^, the variation of annual radon concentrations was up to 13%, agreeing with previous results.

Research on outdoor radon levels in Africa is scarce and in its infancy, revealing considerable research gaps and a lack of thorough assessments of radon exposure risks in comparison to other regions. Although there have been some studies, such as the one conducted by^31^ in South Africa’s West Rand area, which recorded outdoor radon levels ranging from $\:32\:to\:1069\:Bq{m}^{-3}$, these figures are significantly higher than the average outdoor radon levels of approximately$\:\:10\:Bq{m}^{-3}$. The insufficient research and data collection regarding outdoor radon pose a critical public health issue, as although outdoor radon levels are generally low, elevated concentrations can still pose health risks. Remarkably high readings were noted from gold tailings dams. In indoor settings, radon levels reached a peak of $\:\:174\:Bq{m}^{-3},$ surpassing the recommended limit of$\:\:100\:Bq{m}^{-3}.$ Similarly a study conducted in Tanzania by^32^ near the Manyoni uranium deposit and the Mkuju River uranium mine, respectively. The results from many locations show an average of $\:5\:to\:15\:Bq{m}^{-3}$worldwide, except for a few cases. This shows that outdoor radon typically dilutes to extremely low levels quickly and poses no threat Additionally, the seasonal fluctuations of radon gas were explored in Ethiopia by^33^, which showed that concentrations peaked during the cooler winter months and were lowest in the warmer summer months of the year and also highlighted that annual variation of outdoor radon gas concentration was insignificant.

Because of its radioactive characteristics, extended exposure to elevated radon levels can have detrimental effects on one’s health, especially an elevated risk of lung cancer^34^. The radiation that is released can interact with stuff and have a variety of characteristics and energy levels. This could harm living things or change the composition of materials. Radiation leakage indicates that radioactive materials need to be handled with caution and with the appropriate safety measures^35^. Various factors, including radon concentration, exposure duration, and individual susceptibility, might influence the health effects of radon exposure. Long-term radon exposure can raise the risk of lung cancer, especially in enclosed spaces where people spend a lot of time, like homes, workplaces, and schools, according to a review by^36^. Although radon exposure can come from a variety of sources, indoor radon is the most dangerous because, as^37^ notes, it can reach significant concentrations in small areas. Another review by^38^ provided strong evidence that inhaling radon in indoor environments is closely associated with the development of lung cancer and childhood leukemia in patients living in Europe and areas with high radon levels ($\:\ge\:100\:Bq{m}^{-3}$). ^222^Rn is responsible for 3–14% of all deaths caused by lung cancer^39^.

As a naturally occurring radioactive gas, radon gas poses a serious global health risk since it can cause lung cancer when ingested at high concentrations for extended periods. It is crucial to precisely identify and map radon gas distribution for environmental security^40^.

As numerous researchers have reported, geological formations are anticipated to be the primary sources of radon gas in the environment^4^. Dessie town is composed of basaltic and undifferentiated alluvial, elluvial, and lacustrine deposits, according to research by^41^ that omits information on the distribution of radon gas. From the review by^7^, we understood that the uranium concentrations in basalt rock range from 0.1 to 1 ppm. We were motivated to assess and map the radon gas distribution in Dessie town, as we are aware that radon gas is created when radium decays. Our observations of Dessie town’s fast urbanization and population growth raise concerns about potential radon gas exposure and the associated health issues. There were few studies on the distribution and concentration of radon gas in Ethiopia, especially in the Amhara National Regional State’s Dessie town. As a result, there was a crucial knowledge gap about the spatial distribution of radon gas in this area, which made it difficult to implement efficient mitigation techniques and presented possible health hazards to the local population. Consequently, there was a critical knowledge gap regarding the spatial distribution of radon gas in this region, hindering effective mitigation strategies and posing potential health risks to the local population. Consequently, developing an effective approach for creating a radon gas distribution map is necessary by integrating geological and geospatial techniques for the security of the environment^42^. Addressing the lack of comprehensive radon gas data in Dessie town using geological and geospatial techniques is crucial for assessing the extent of health risks and developing evidence-based mitigation strategies. By bridging this gap, this research aims to contribute to the scientific understanding of radon gas distribution in Ethiopia and facilitate informed decision-making for public health protection. In locations where radon gas concentrations are higher, there is a significant risk of radon exposure. While geology is the primary source of radon, its concentration, movement, and accumulation are heavily influenced by a range of non-geological factors (meteorological conditions, building characteristics, and groundwater). In a rapidly urbanizing town like Dessie, the interplay between geology and non-geological factors is particularly significant. In this study, geological formations and geospatial methods were used to estimate and map the radon gas distribution in Dessie town.

## Materials and methods

### Study area

**Fig. 1:**
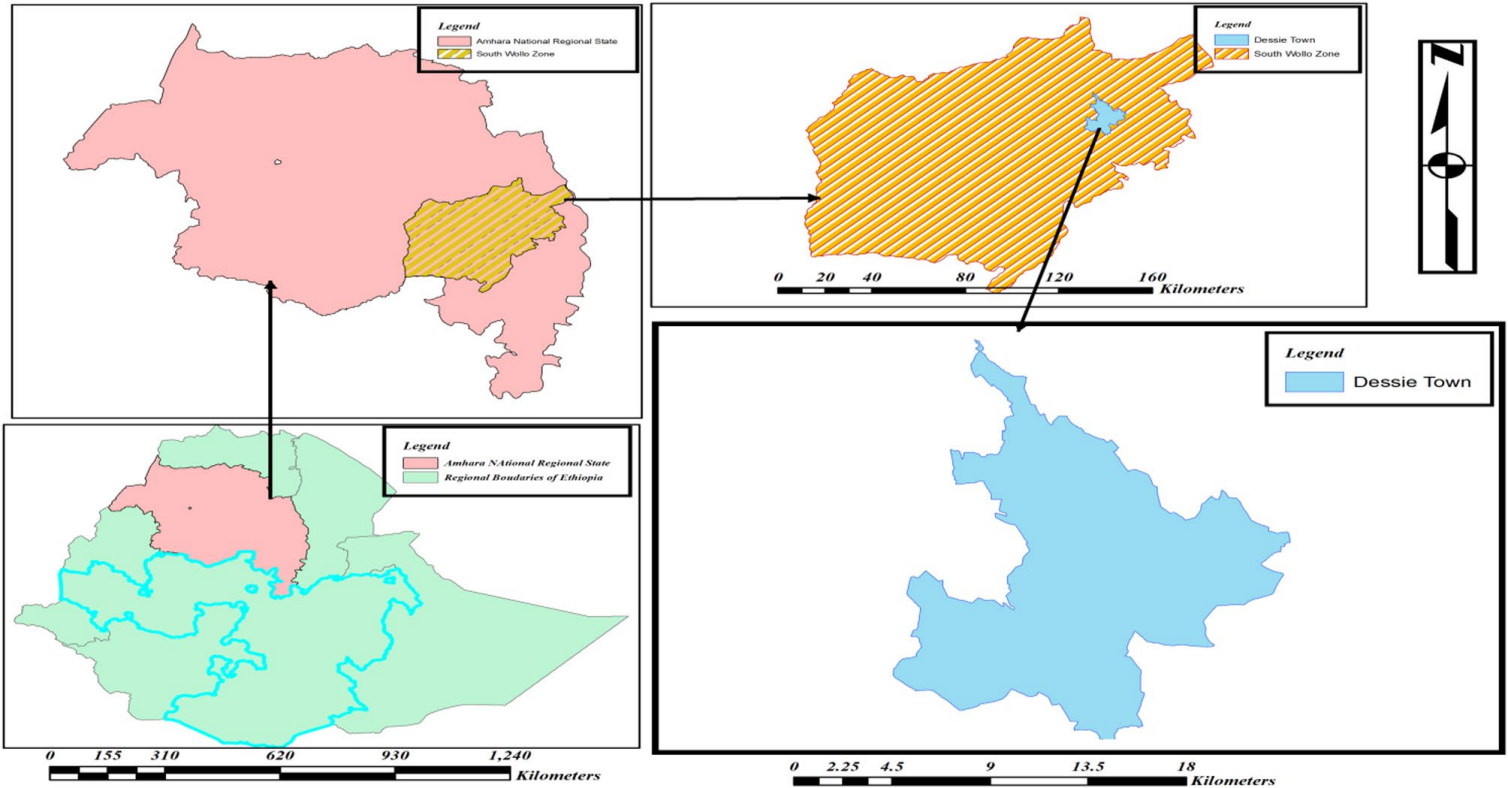
Map of the study area. Source (QGIS 3.34.0).

The study was conducted at Dessie town, which is a town in north-central Ethiopia. It is located in the South Wollo Zone of the Amhara Region, at a latitude and longitude of 11°8′N 39°38′E, with an elevation between 2,470 and 2,550 m above sea level, as shown in Fig. 1 below. Dessie town is located 400 km north of the capital city of Ethiopia, Addis Ababa. According to a report from the vital statistics office, the total population is approximately 385,850. This population is divided into 26 kebeles—18 urban and eight rural. The scale of the map for Ethiopia, the Amhara region, and the south Wollo zone is 1:2,000,000 and the Dessie town map is 1:200,000, as indicated in the scale bar in the figure below.

Since the study was conducted in Dessie town by using geological and geospatial analysis methods. The geospatial data(Soil moisture, specific humidity, wind speed, and precipitation data) was taken from the source^43^ by using the longitudinal and latitudinal location of Dessie town. As a result of this longitudinal and latitudinal location, there exist eight station points that are included in this address. So, for our study, we had taken eight station points with one data point per station.

### Method of data collection

We gathered geological data, including maps, reports, and geological surveys of the study area from the Ethiopian Geological Survey (EGS) to identify rock types that serve as the source of radon gas, and we have taken shape files for the study area to mask with its geological map. Geo-spatial data was taken from the source^43^ for the study area. For our study area, the Dessie town map was sourced from the library of the Ethiopian Geological Institute.

### Method of data analysis

Geological Analysis: To assess potential sources and pathways of radon gas emissions, we analyzed geological maps and reports by masking shape files and their geological maps with QGIS, which clearly shows rock types placed in this study area. We evaluated rock types that have a high concentration of uranium.

Geospatial analysis: Geospatial analysis was conducted on satellite-derived climate factor data. To combine geological and geospatial data layers, use Quantum Geographic Information Systems (QGIS) software. Use spatial analysis to determine regions with a higher risk of radon gas buildup based on geological and environmental factors.

We implement statistical analysis to get the annual mean, standard deviation, Standard Error of the Mean (SEM), and error bars of climate data (Soil moisture, humidity, wind speed, and precipitation data) values for each year, and which helps to decide the radon gas distributions since they are factors for radon gas concentration amounts.

To determine radon activity concentrations from uranium activity concentrations, we used the following equations:-.

The concentrations of uranium in the identified rock type were determined in ppm, or $\:{Bqkg}^{-1}$ then converted this to $\:{Bqm}^{-3}$ using the conversion factor given below^44^:

1 $$\begin{gathered} {\text{Activity concentrations of uranium in}}\,\,Bq{m^{ - 3}}\,{\text{in IDentified rock type}}\, \hfill \\ =\,{\text{Activity concentrations of uranium in }}Bqk{g^{ - 1}}*\,\,{\text{Density of IDentified rock type in}}\,\,kg{m^{ - 3}} \hfill \\ \end{gathered}$$

After that uranium activity concentration in $\:{Bqm}^{-3}$ was obtained and the radon gas activity concentration calculated in $\:{Bqm}^{-3}$ by using the conversion factor below^45^:

2 $$\begin{gathered} {\text{Radon gas activity concentrations }}\left( {{\text{RAC}}} \right){\text{ in}}\,Bq{m^{ - 3}}={\text{ Uranium activity concentration }}\left( {{\text{UAC}}} \right){\text{ in}}\,\,Bq{m^{ - 3}} \hfill \\ *\,\,~\left( {{\text{Radon emanation coefficient}}} \right){\text{ E}} \hfill \\ \end{gathered}$$

The percentage of radon that escapes from the geological formation (rock and soil) into the atmosphere is represented by the radon gas emanation coefficient, or E. According to data from the International Atomic Energy Agency, basalt and other thick minerals often have emanation coefficients that are on the lower end of the spectrum (0.1 to 0.3).

## Result and discussion

### Identifications of the geological formation

**Fig. 2:**
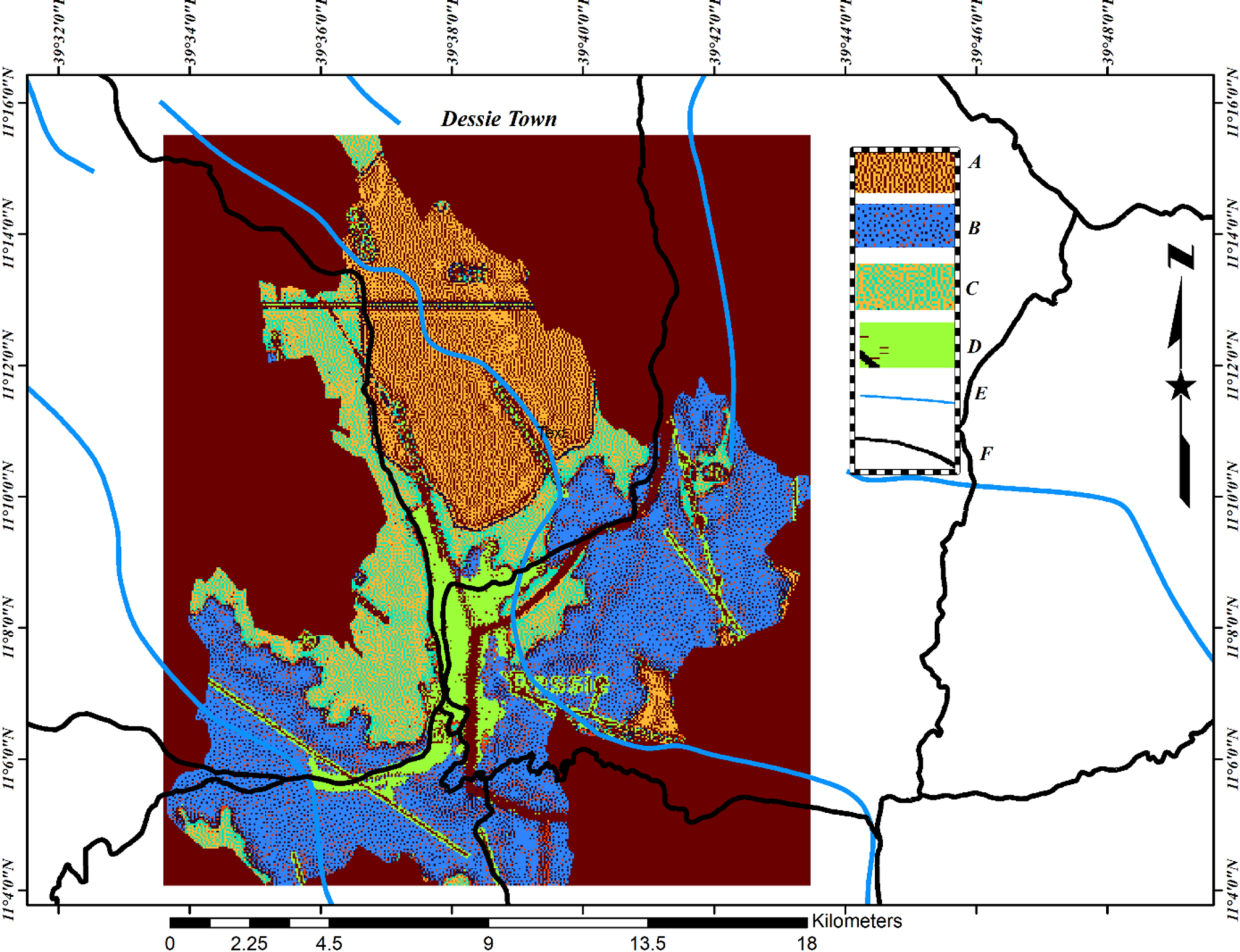
Maps showing geological formations of Dessie town, **‘A’** represents undifferentiated alluvial, elluvial, and lacustrine sediment, **‘B’** represents Ashangi basalt, **‘C’** represents Dessie basalt formation, **‘D’** represents the main town (basalt ), **‘E’** represents rivers, and **‘F’** represents roads. Source (Ethiopian Survey Agency, 1997).

The geological structures of Dessie town consist of basalt rocks, as indicated on the Dessie town map sheet included in the appendix. The Dessie town map was drawn using QGIS software version 3.34, as illustrated in Fig. 2 below, to depict its geological formations. By utilizing the map sheet and shape files for Dessie, we identified the geological formations present in Dessie town as detailed below.

### Climatic factors that influence radon gas distributions

As previously mentioned, the levels of radon gas and its movement are affected by climate conditions. Consequently, we have included precipitation, wind speed, specific humidity, and surface soil moisture in our analysis of the study area. The information we utilized for these climatic variables was sourced from^43^ for the years 2020, 2021, and 2022 consecutively. Our study area, Dessie town, is located within a latitude range $\:{11.067}^{0}\:and\:{11.267}^{0}\:N\:\:$and a longitude range$\:\:{39.53}^{0}\:and\:{39.73}^{0}\:E$. So, the adopted climate factor satellite data was based on this latitude and longitude range for eight different stations in the study area.

### Surface soil moisture

Soil moisture affects the permeability and diffusion capacity of radon. Wet soil can limit the mobility of radon, acting as a barrier to radon gas diffusion; as a result of this, the radon gas concentrations become lower. The soil moisture content data of Dessie town in eight (8) different stations, latitude ($\:{10.25}^{0}$,$\:\:{10.75}^{0},\:{11.25}^{0}\:and\:{11.25}^{0}$), long ($\:{39.25}^{0}$and$\:\:{39.75}^{0}$) for three consecutive years from 2020 to 2022, as adopted from^43^, is shown in the figure below.

**Fig. 3:**
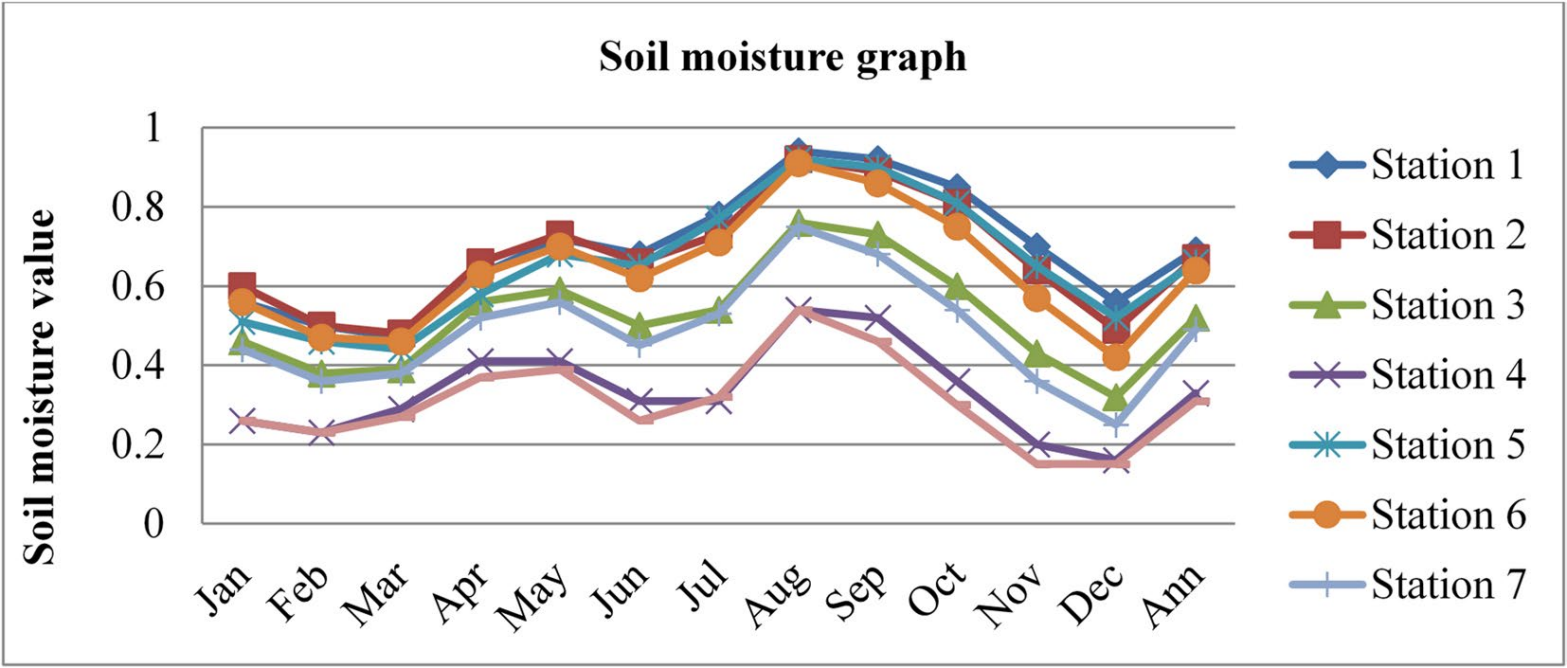
Soil moisture content of Dessie town in 2020. Source^43^

**Fig. 4:**
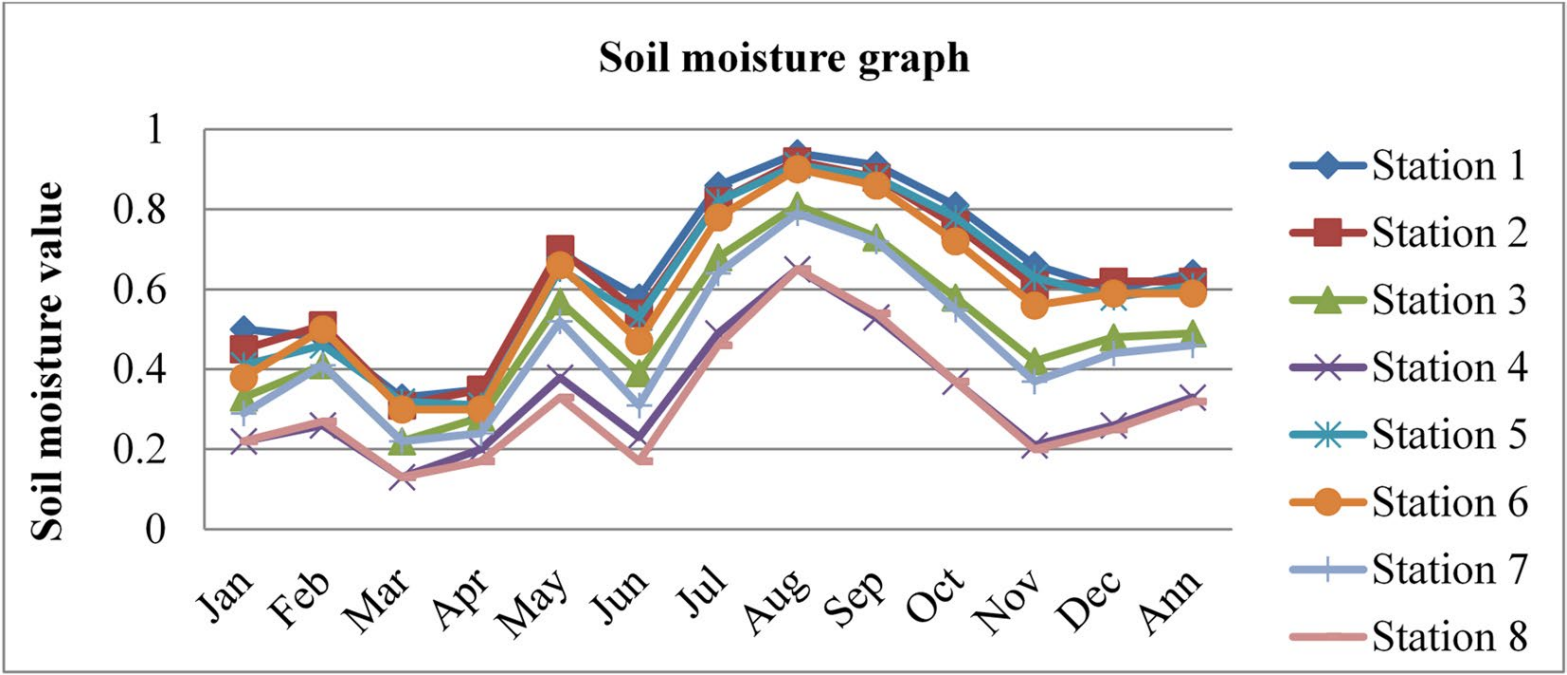
Soil moisture content of Dessie town in 2021. Source^43^

**Fig. 5:**
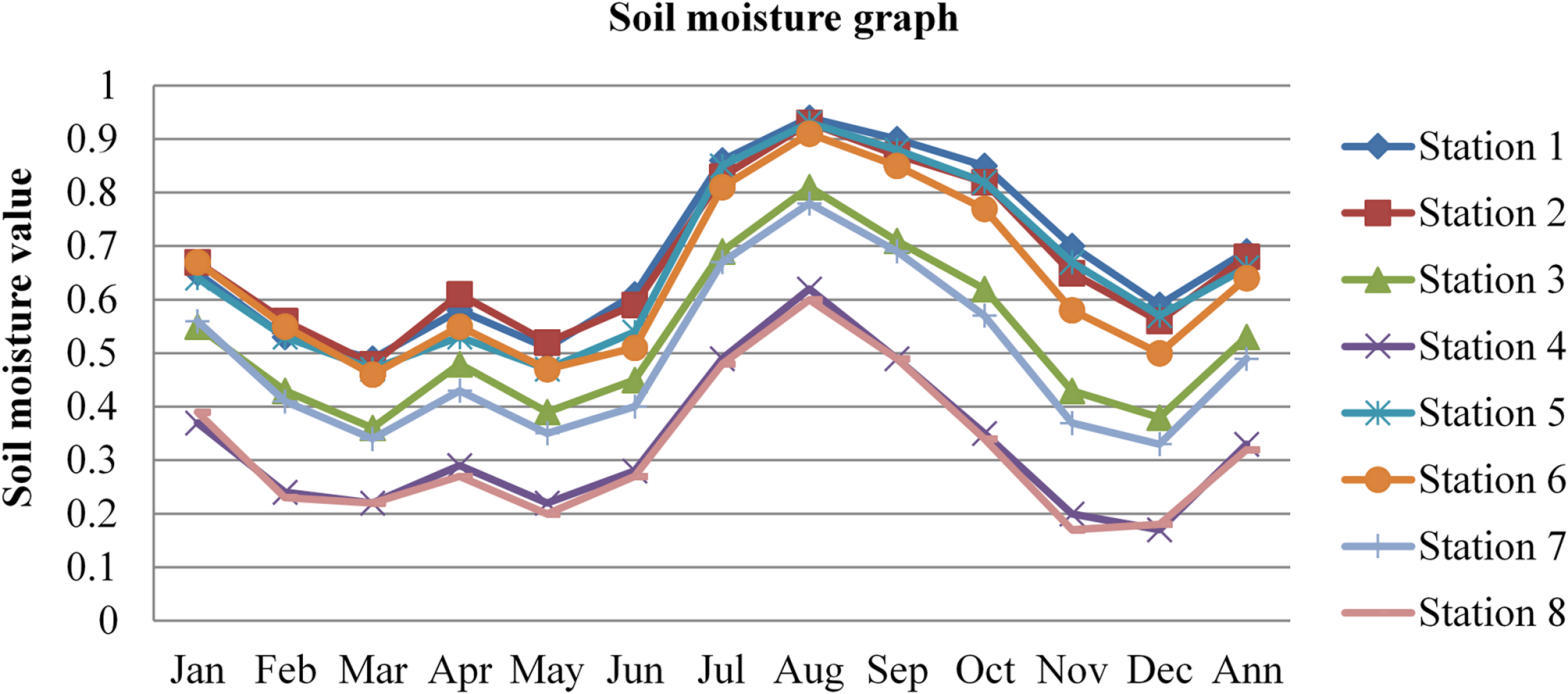
Soil moisture content of Dessie town in 2022. Source^43^

From the above three graphs (Figs. 3 and 4, and Fig. 5), we observed that for three consecutive years, the highest values were generally recorded between July and September (Ethiopia’s kiremt season), corresponding to lower radon concentrations. Conversely, the lowest soil moisture levels occurred between December and February (Ethiopia’s Bega season), associated with higher radon concentrations, which agree with results reported by^28,29^;^33^. The lowest values of outdoor radon gas concentrations were in winter (Kiremt in Ethiopia), presumably due to a snow cover, large humidity, or strong winds. This happens because moist soil tends to have smaller pore spaces filled with water, reducing the movement of radon through the soil^19^. This can lead to lower radon concentrations in the outdoor environment.

**Fig. 6:**
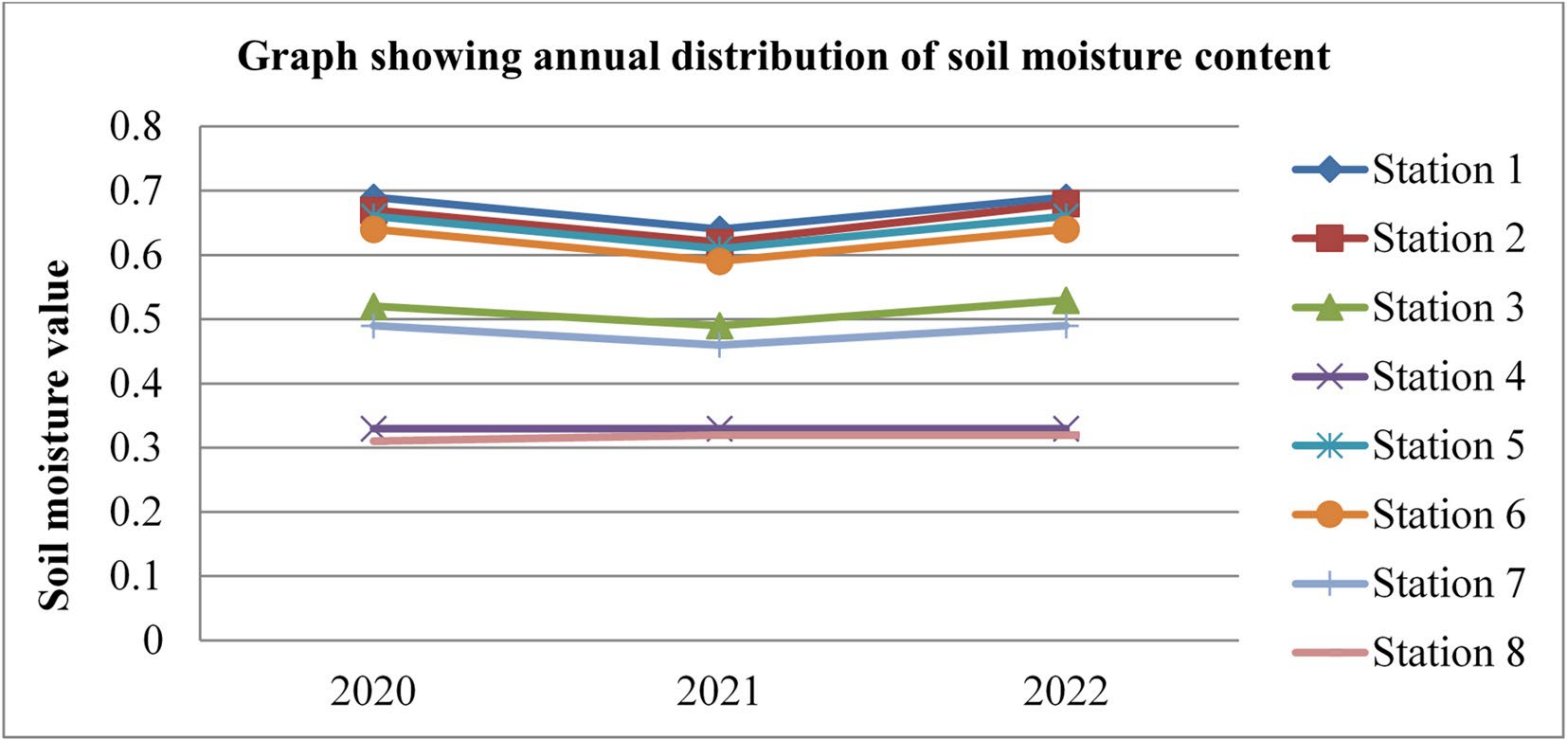
Annual soil moisture content of Dessie town from 2020–2022. Source^43^

Based on the annual soil moisture content data for Dessie town from the eight stations, here are the standard deviations and the corresponding error bars for each of the three years. The error bars represent the standard error of the mean (SEM), which is calculated as the standard deviation divided by the square root of the number of stations (*n* = 8).

**Table 1:** Soil moisture content statistical indicator of Dessie town from 2020–2022.

Statistical indicator	Years		
	2020	2021	2022
Mean	0.539	0.508	0.543
Standard deviation (SD)	0.153	0.129	0.152
Standard error of the mean (SEM)	0.054	0.046	0.054
Error bars (mean ± SEM)	0.539 ± 0.054	0.508 ± 0.046	0.543 ± 0.054

Source^43^.

From Fig. 6; Table 1, we saw variations in soil moisture content for three consecutive years at each station and its statistical indicator result almost similar. Station 1 exhibited the highest values with 0.69%, 0.64%, and 0.69% in 2020, 2021, and 2022, respectively. Station 8 had the lowest values at 0.31%, 0.32%, and 0.32% for the same period. This shows that there is a small annual variation in soil moisture content, which gives a small annual variation in radon gas. The soil moisture variation is similar to radon gas concentration variations, and the result of the annual variation of radon gas is small. This result agree with previous results obtained like in 4 years of continuous monitoring in Milan, Italy conducted by^24^ variation of annual radon concentrations was up to 13% and a study conducted by^30^ found that the annual average outdoor radon concentration measured for a few consecutive years did not show significant ($\:<15\%$) differences between annual concentrations. Another finding, also conducted in Slovenia by^10^ and in Ethiopia by^33^, shows no significant difference between outdoor radon concentrations in any of the 4 seasons (annual).

### Specific humidity

Specific humidity also affects the concentrations and mobility of radon gas. Higher humidity levels can enhance the mixing and dilution of radon in the outdoor atmosphere. This dilution effect occurs as moisture-laden air mixes with the surrounding air, potentially reducing the overall concentration of radon gas. The specific humidity data adopted from^43^ for three consecutive years (2020, 2021, and 2022) was given in the figures below.

**Fig. 7:**
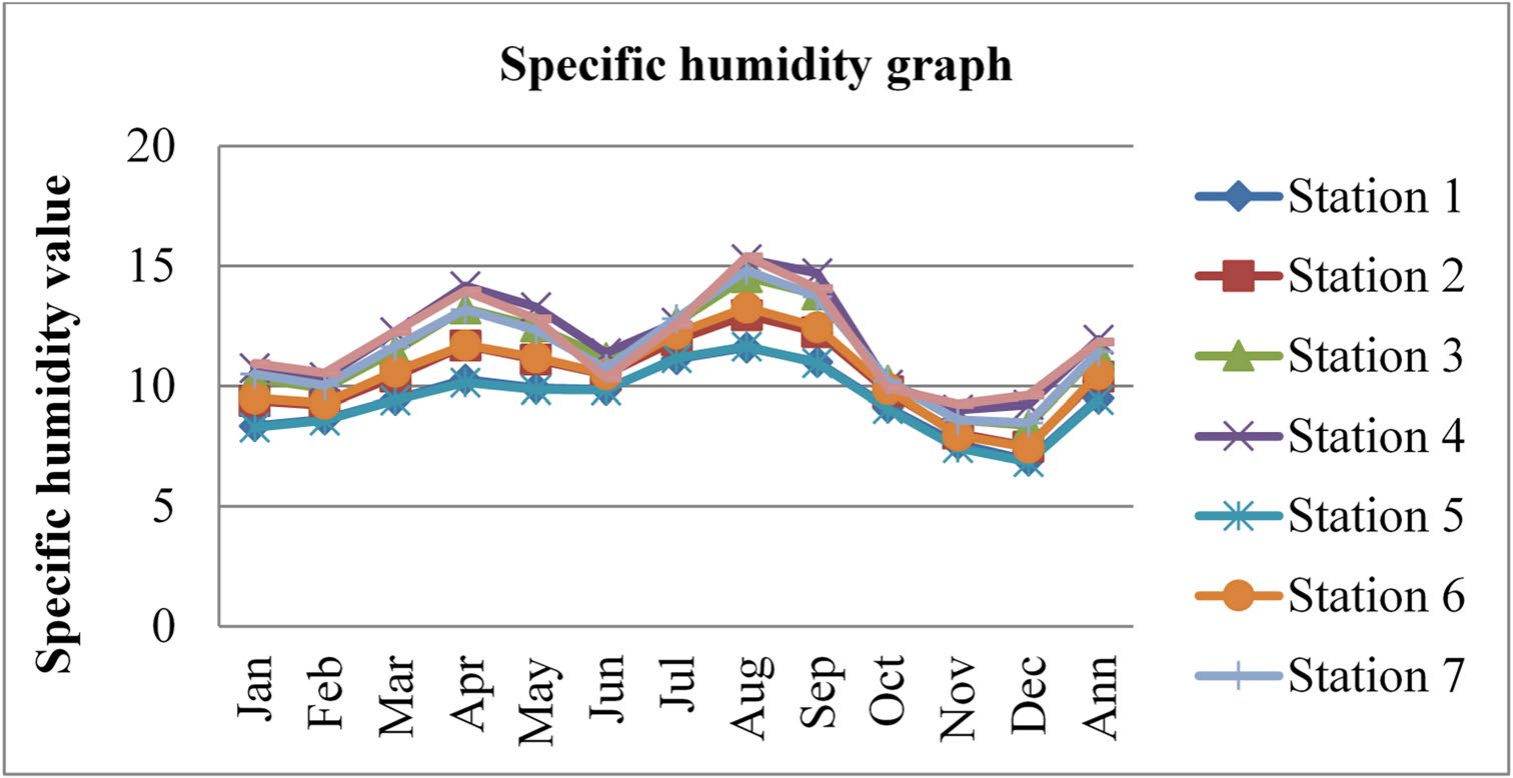
Specific humidity of Dessie town in 2020. Source^43^

**Fig. 8:**
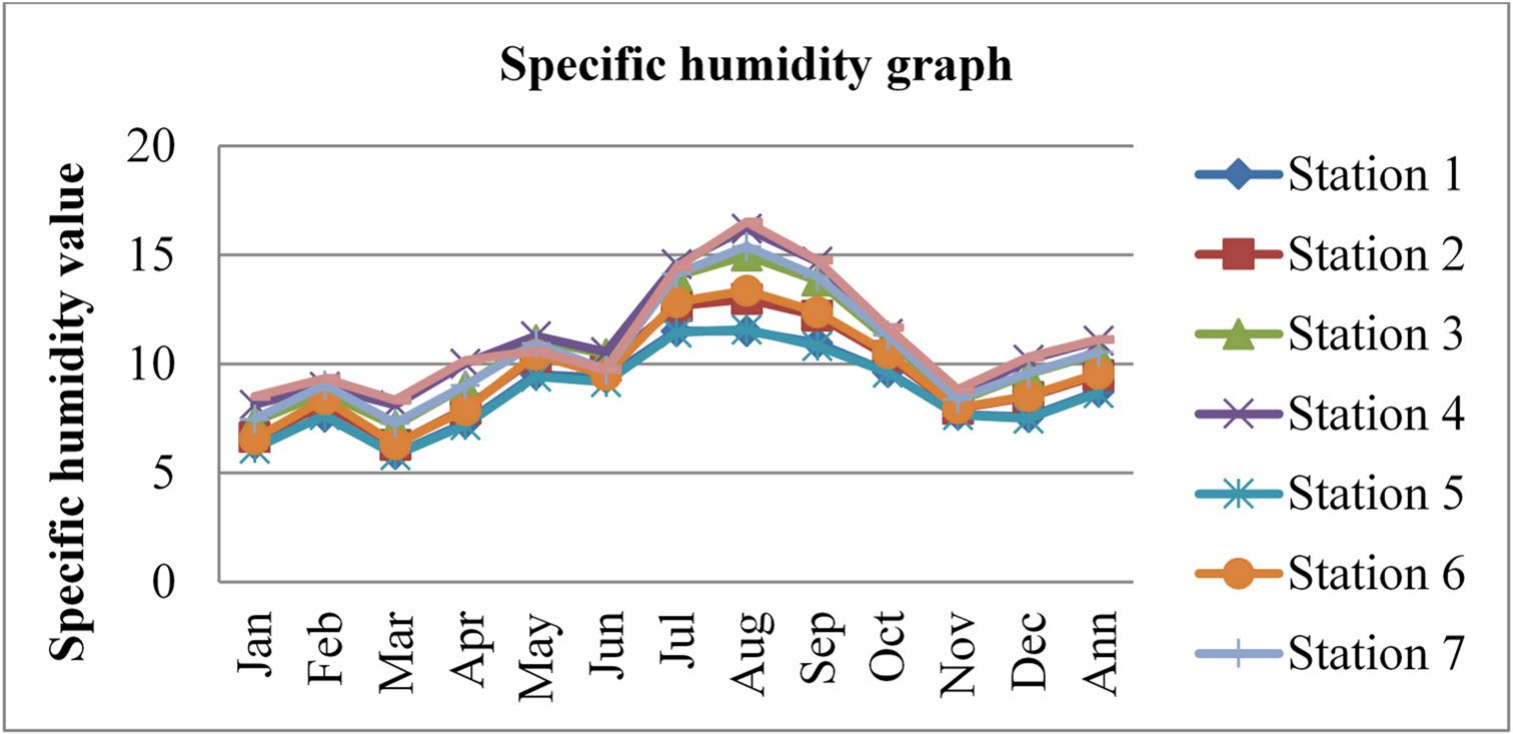
Specific humidity of Dessie town in 2021. Source^43^

**Fig. 9:**
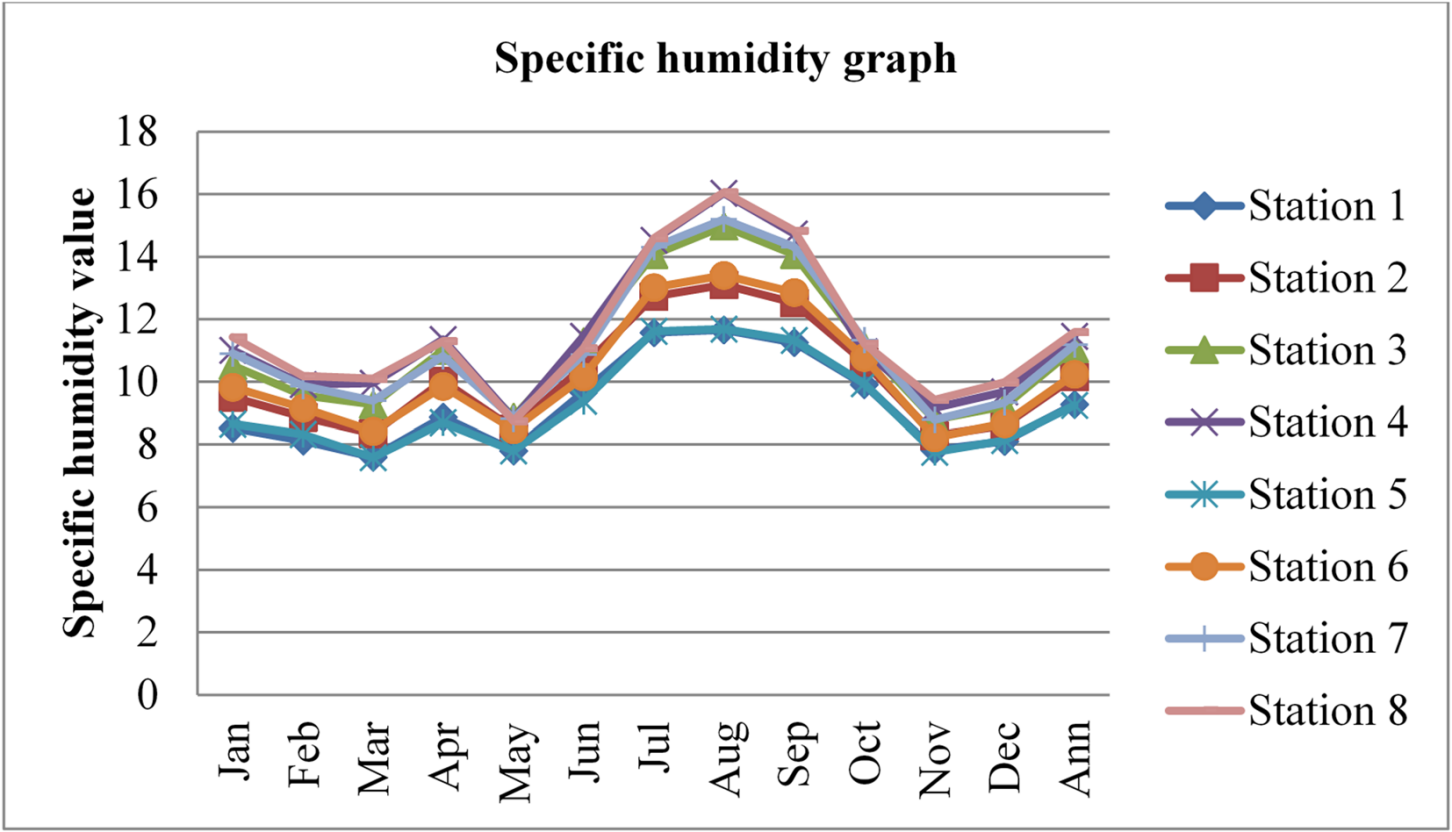
Specific humidity of Dessie town in 2022. Source^43^

From the above three graphs (Figs. 7, 8 and 9) we observed that the specific humidity become greater at all stations in summer (kiremt season in Ethiopia) July, August and September which results low radon gas concentrations and the minimum value was observed at all stations in winter (Bega season in Ethiopia) December, January and February some times in March and in November which results in high radon gas concentrations, which agree with results reported by^28,29,33^ the lowest values of outdoor radon gas concentrations were in winter (Kiremt in Ethiopia), presumably due to a snow cover, large humidity, or strong winds. Radon concentrations fall as specific humidity increases, as radon gas diffuses more slowly in moist air compared to dry air. Higher specific humidity can hinder the movement of radon through the air, resulting in slightly lower outdoor radon concentrations as reported by^21^.

**Fig. 10:**
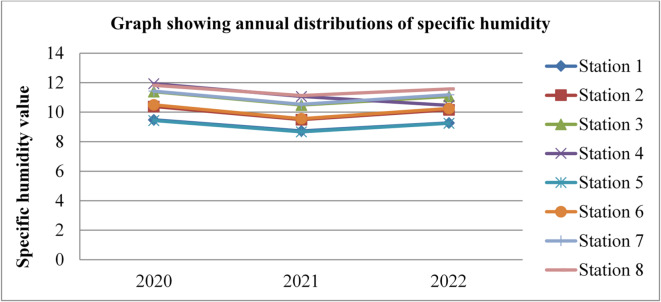
Annual specific humidity of Dessie town from 2020–2022. Source^43^

Based on the annual specific humidity data for Dessie town from the eight stations, here are the standard deviations and the corresponding error bars for each of the three years.

The error bars represent the standard error of the mean (SEM), which is calculated as the standard deviation divided by the square root of the number of stations (*n* = 8).

**Table 2:** Specific humidity statistical indicator of Dessie town from 2020–2022.

Statistical indicator	Years		
	2020	2021	2022
Mean	10.799	9.960	10.403
Standard deviation (SD)	0.992	0.982	0.856
Standard error of the mean (SEM)	0.350	0.347	0.303
Error bars (mean ± SEM)	10.799 ± 0.350	9.960 ± 0.347	10.403 ± 0.303

Source^43^.

From Fig. 10; Table 2, we understand that the annual variations of specific humidity at each station for three consecutive years are almost similar, which implies that the annual concentrations of radon gas are constant or the variations in radon gas concentrations annually are almost small for each station and its statistical indicator result almost similar. This shows that there is a small annual variation of specific humidity, which gives a small annual variation of radon gas. The specific humidity variation is similar to that of radon gas concentration variations, and gives the result that the annual variation of radon gas is small. This result agree with previous results obtained like in 4 years of continuous monitoring in Milan, Italy conducted by^24^ variation of annual radon concentrations was up to 13% and a study conducted by^30^ found that the annual average outdoor radon concentration measured for a few consecutive years did not show significant ($\:<15\%$) differences between annual concentrations. Another finding, also conducted in Slovenia by^10^ and in Ethiopia by^33^, shows no significant difference between outdoor radon concentrations in any of the 4 seasons (annual).

### Wind speed

Wind speed affects radon gas distributions and mobility. Higher wind speeds facilitate the dispersion and transport of radon gas, leading to lower radon gas concentrations in the outdoor environment and potentially reducing the risk of radon exposure. The wind speed data was adopted from^43^ for three consecutive years, 2020, 2021, and 2022, and is given in the figures below.

**Fig. 11:**
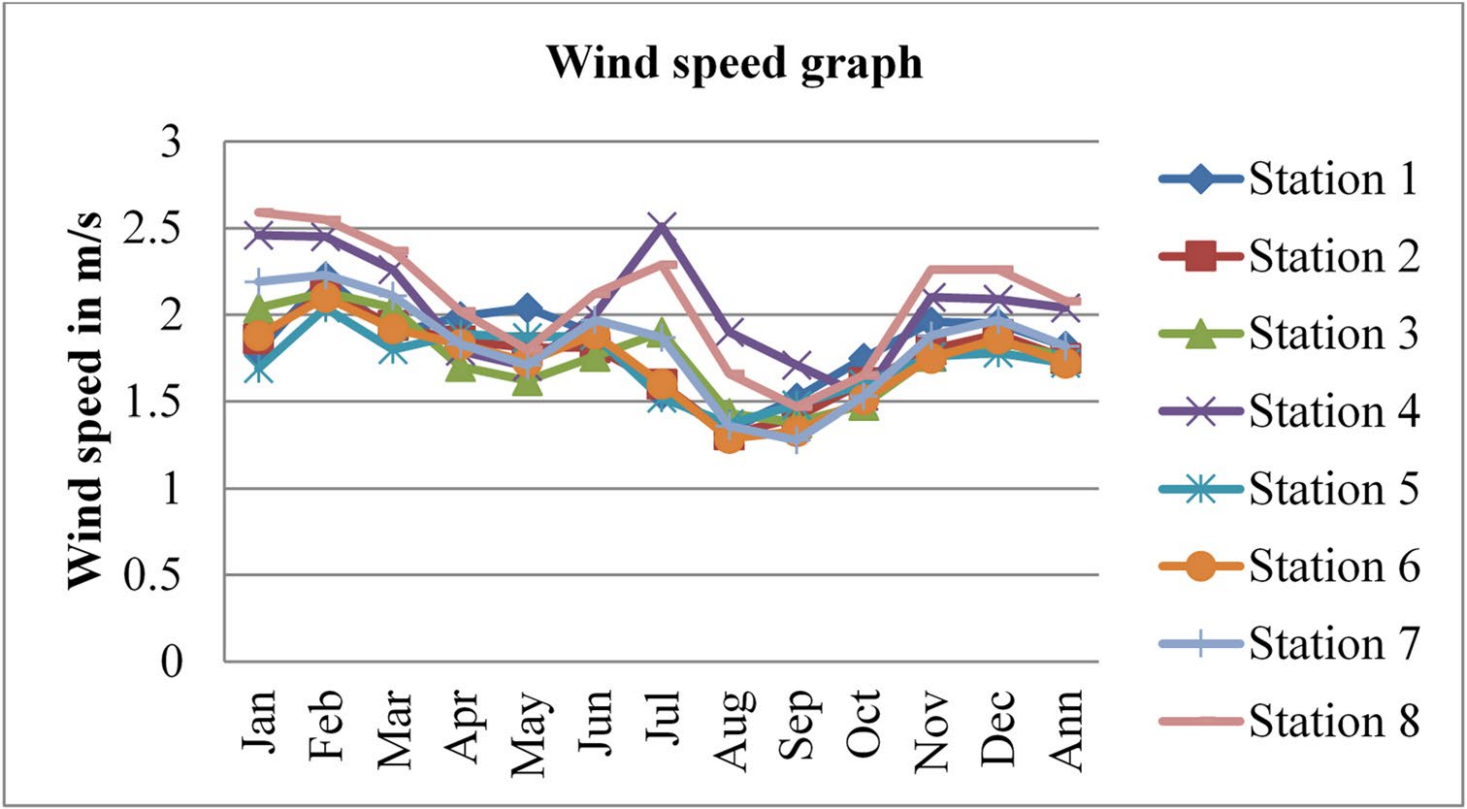
Wind speed in Dessie town in 2020. Source^43^

**Fig. 12:**
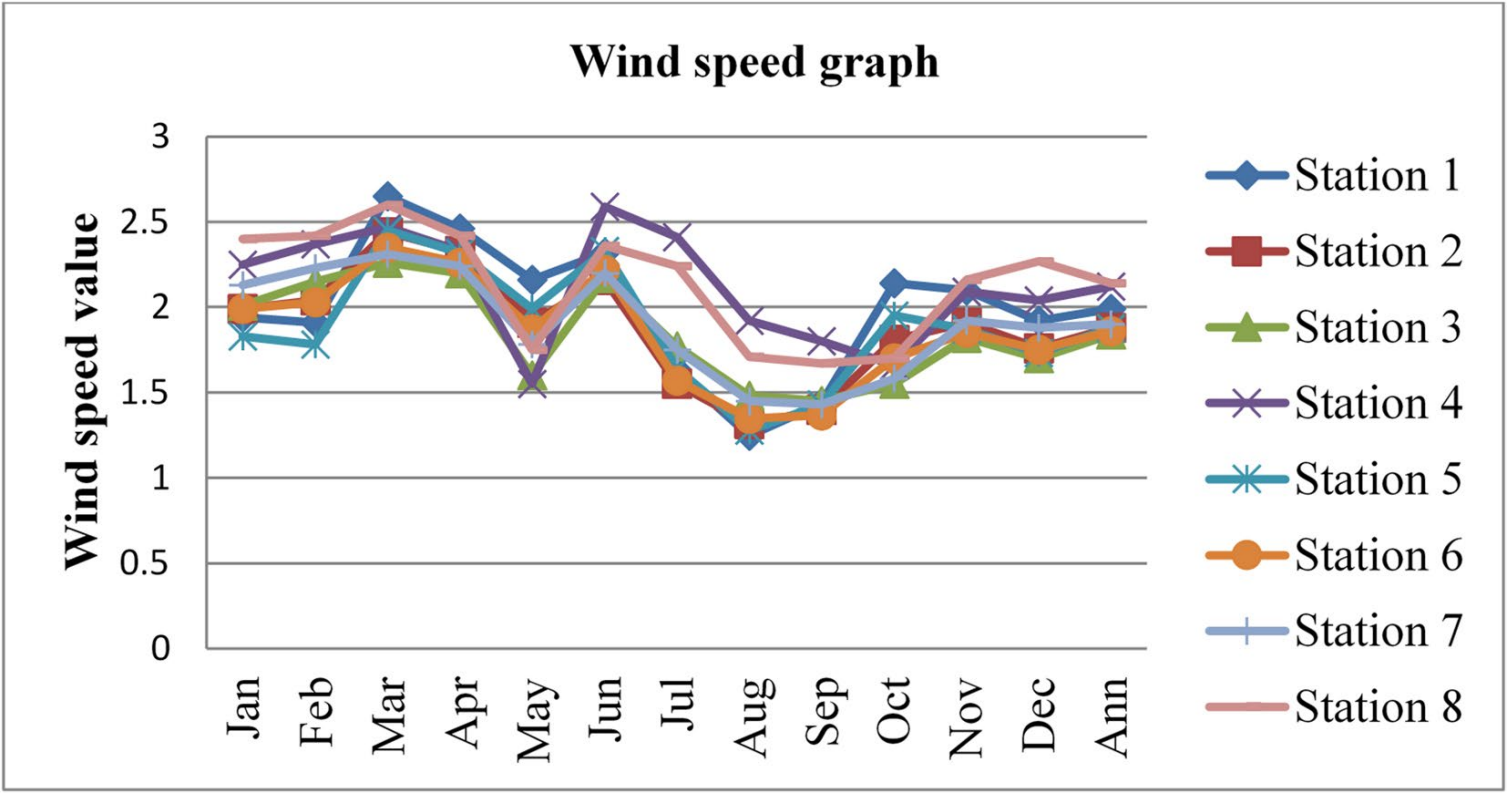
Wind speed of Dessie town in 2021. Source^43^

**Fig. 13:**
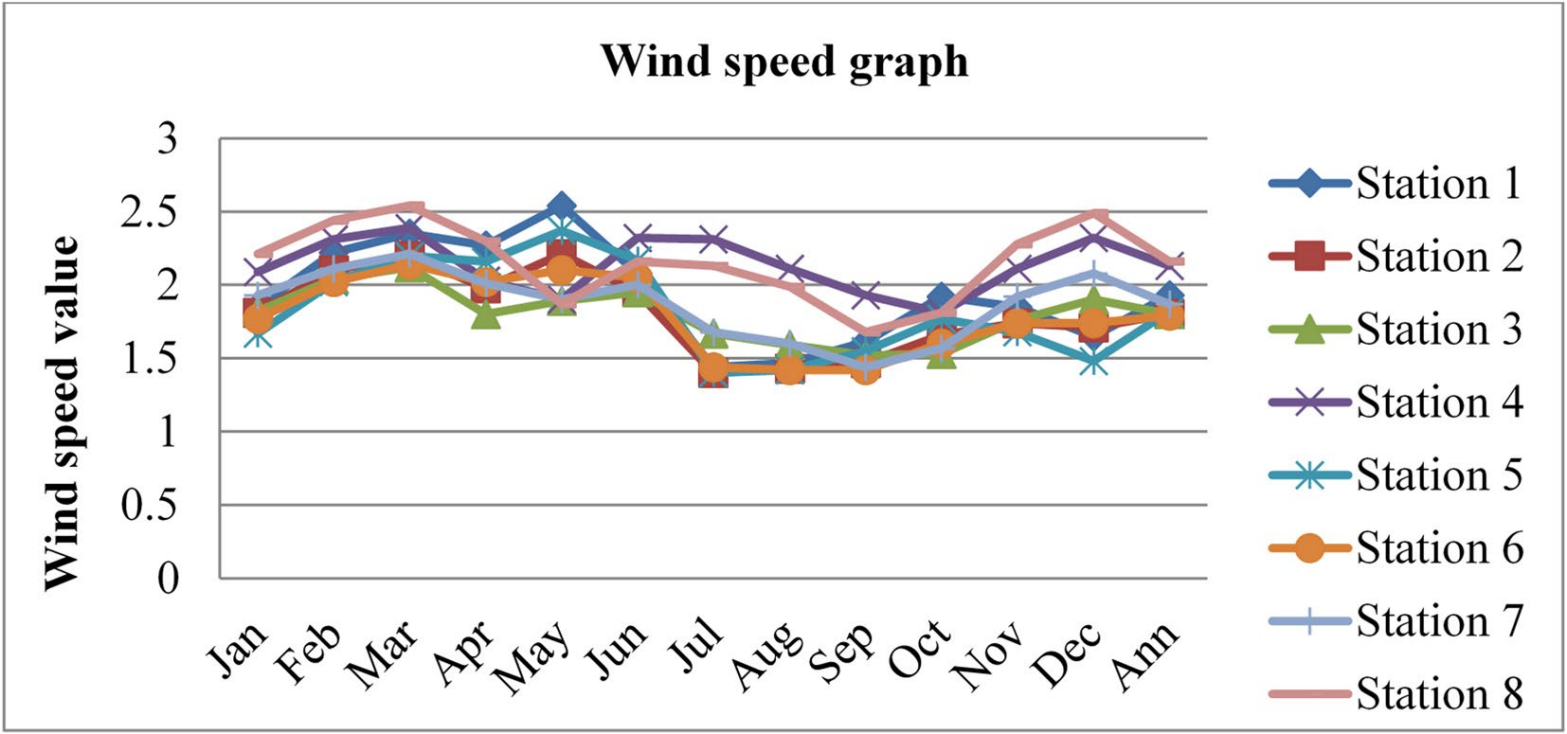
Wind speed of Dessie town in 2022. Source^43^

From the above three graphs (Figs. 11, 12 and 13) we understand the wind speed most commonly become greater at all stations in January, February and March which implies that low radon gas distributions in these seasons and become minimum most commonly in August, September and October which implies that high radon gas distributions in these seasons. This result shows that low radon concentrations occur when wind speed increases, and high radon gas concentrations occur when wind speed decreases, as reported by^46^. Outdoor wind speed and relative humidity show a negative correlation with both outdoor and indoor radon concentrations at all locations.

**Fig. 14:**
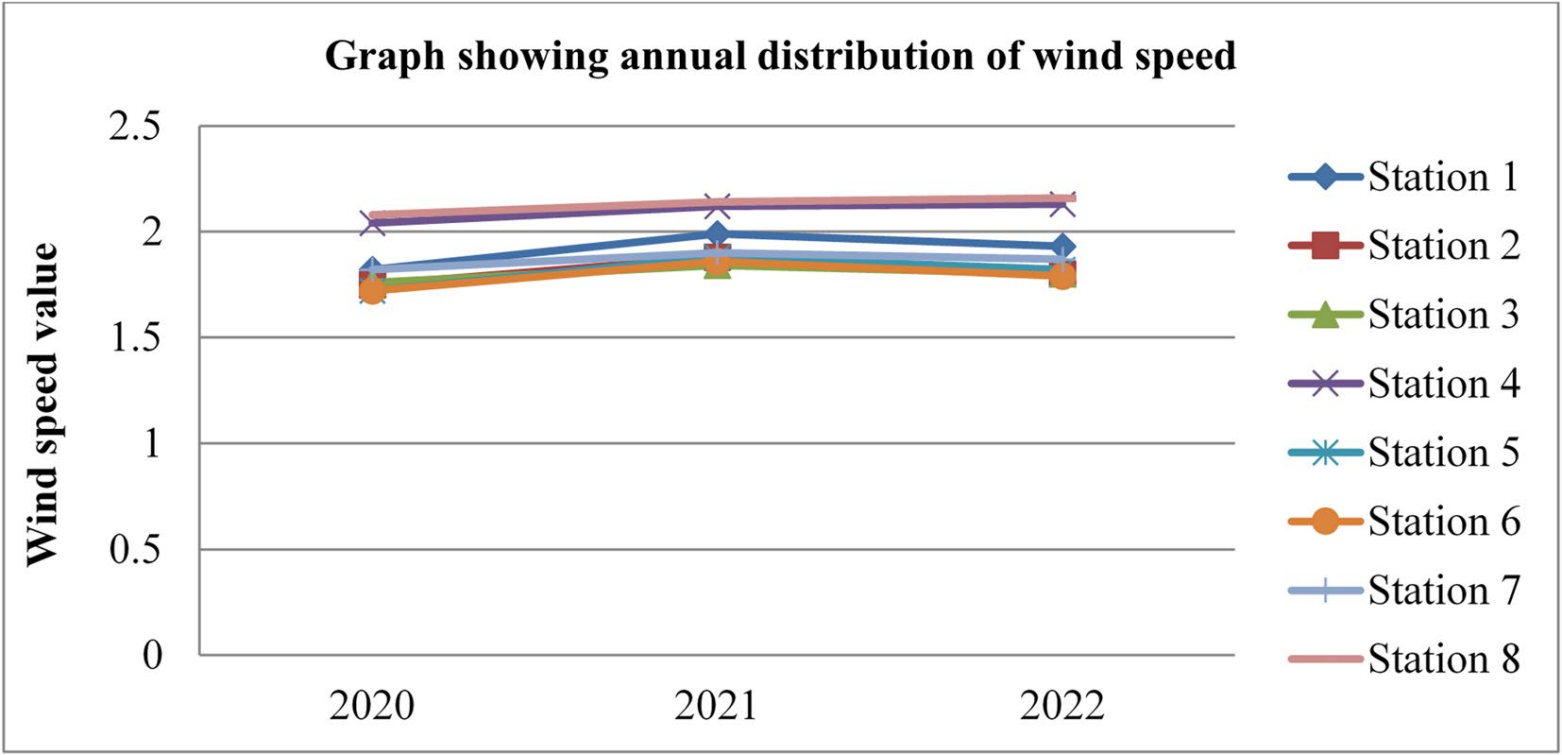
Annual wind speed of Dessie town from 2020–2022. Source^43^

Based on the annual wind speed data for Dessie town from the eight stations, here are the standard deviations and the corresponding error bars for each of the three years.

The error bars represent the standard error of the mean (SEM), which is calculated as the standard deviation divided by the square root of the number of stations (*n* = 8).

**Table 3:** Wind speed statistical indicator of Dessie town from 2020–2022.

Statistical indicator	Years		
	2020	2021	2022
Mean	1.839	1.951	1.913
Standard deviation (SD)	0.142	0.119	0.152
Standard error of the mean (SEM)	0.050	0.042	0.053
Error bars (mean ± SEM)	1.839 ± 0.050	1.951 ± 0.042	1.913 ± 0.053

Source^43^.

From Fig. 14; Table 3, we saw annual variations of wind speed for three consecutive years at each station. And its statistical indicator result almost similar. This result shows that there is a small annual variation in wind speed, which gives a small annual variation in radon gas. The wind speed variation is similar to radon gas concentration variations, and suggests that the result of the annual variation of radon gas is small. This result agree with previous results obtained like in 4 years of continuous monitoring in Milan, Italy conducted by^24^ variation of annual radon concentrations was up to 13% and a study conducted by^30^ found that the annual average outdoor radon concentration measured for a few consecutive years did not show significant ($\:<15\%$) differences between annual concentrations. Another finding, also conducted in Slovenia by^10^ and in Ethiopia by^33^, shows no significant difference between outdoor radon concentrations in any of the 4 seasons (annual).

### Precipitations

Precipitation affects radon gas distributions and mobility. Precipitation can act as a natural washout mechanism for radon gas. As rain or snowfall occurs, radon molecules can be scavenged from the air and deposited onto the ground or other surfaces. This washout effect can reduce radon concentrations in the outdoor environment temporarily. The precipitation data adopted from^43^ for three consecutive years, 2020, 2021, and 2022, is given in the figures below.

**Fig. 15:**
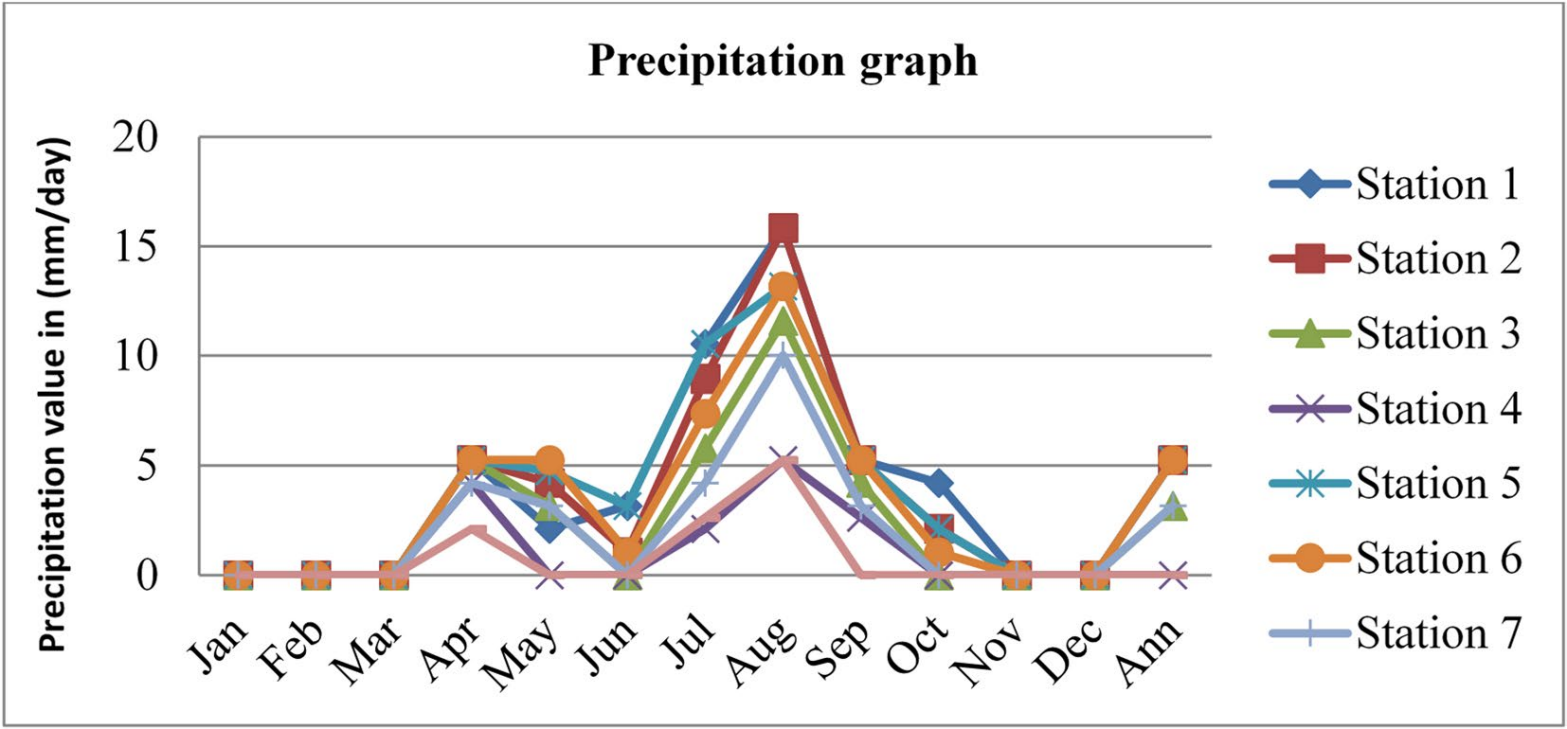
Precipitation of Dessie town in 2020. Source^43^

**Fig. 16:**
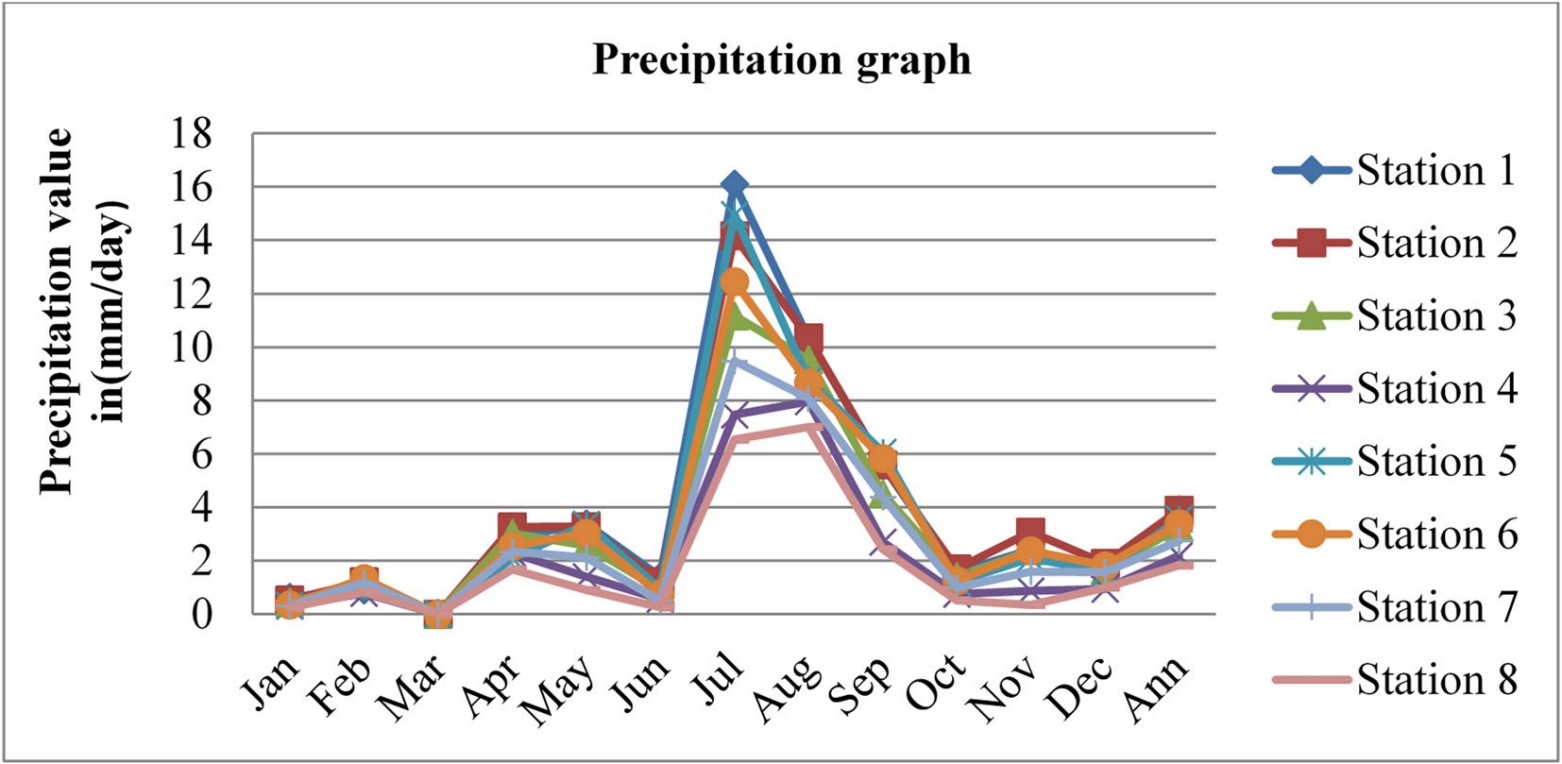
Precipitation of Dessie town in 2021. Source^43^

**Fig. 17:**
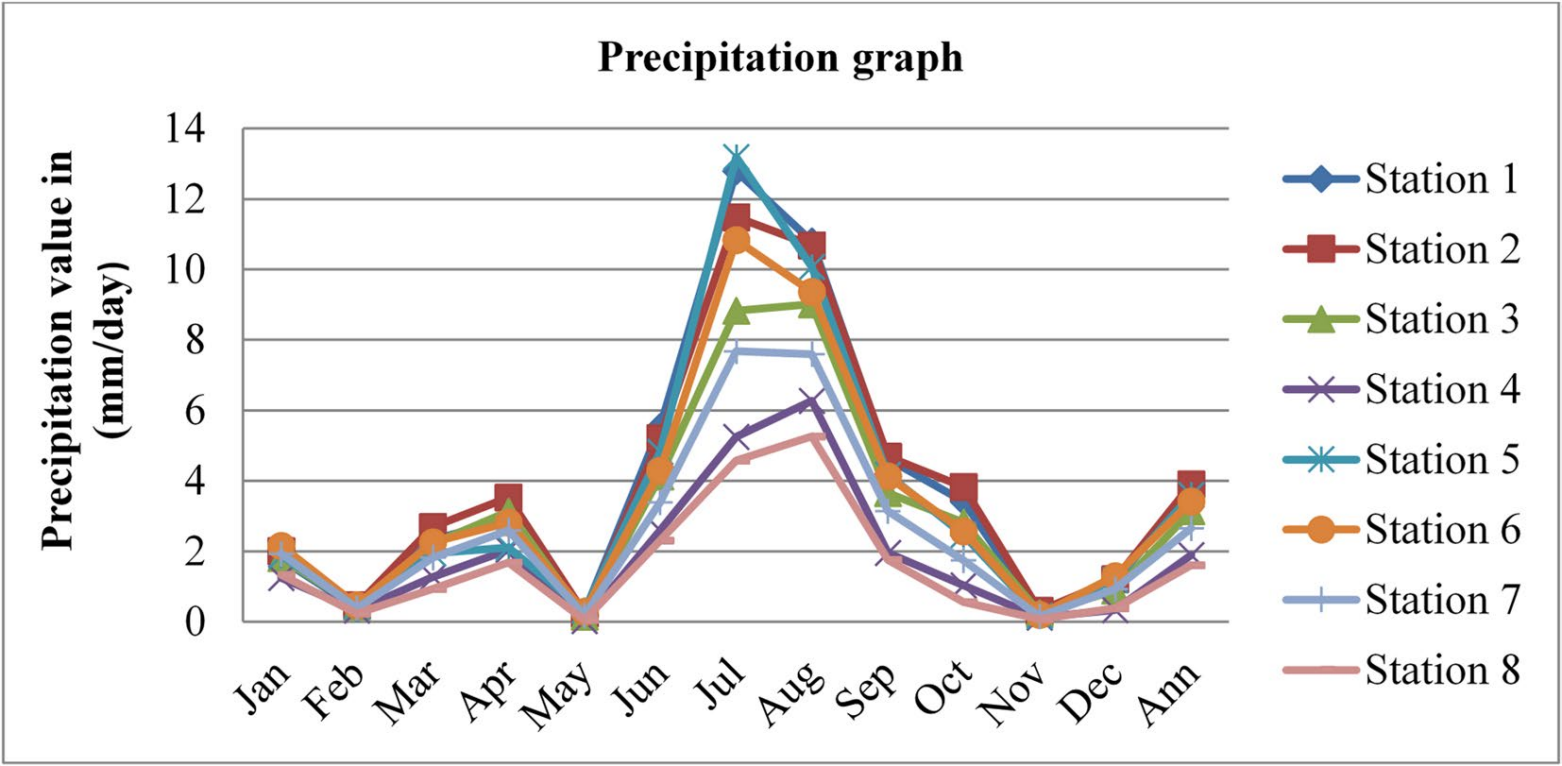
Precipitation of Dessie town in 2022. Source^43^

From the above three graphs (Figs. 15 and 16, and Fig. 17) we understand the precipitation most commonly become greater at all stations in July and August which implies that low radon gas distributions in these seasons and become minimum most commonly in December, January, February and March which implies that high radon gas distributions in these seasons. As high precipitation decreases radon gas concentrations and low precipitation increases radon gas concentrations, as reported by^47^, increased precipitation acts to impede radon emanation, “capping” the soil outdoors and directing it toward the unsaturated soil near or under the building. In addition, if the soil is not saturated, low and moderate levels of soil moisture provide a greater radon source that can penetrate through holes in the substructure of a building.

**Fig. 18:**
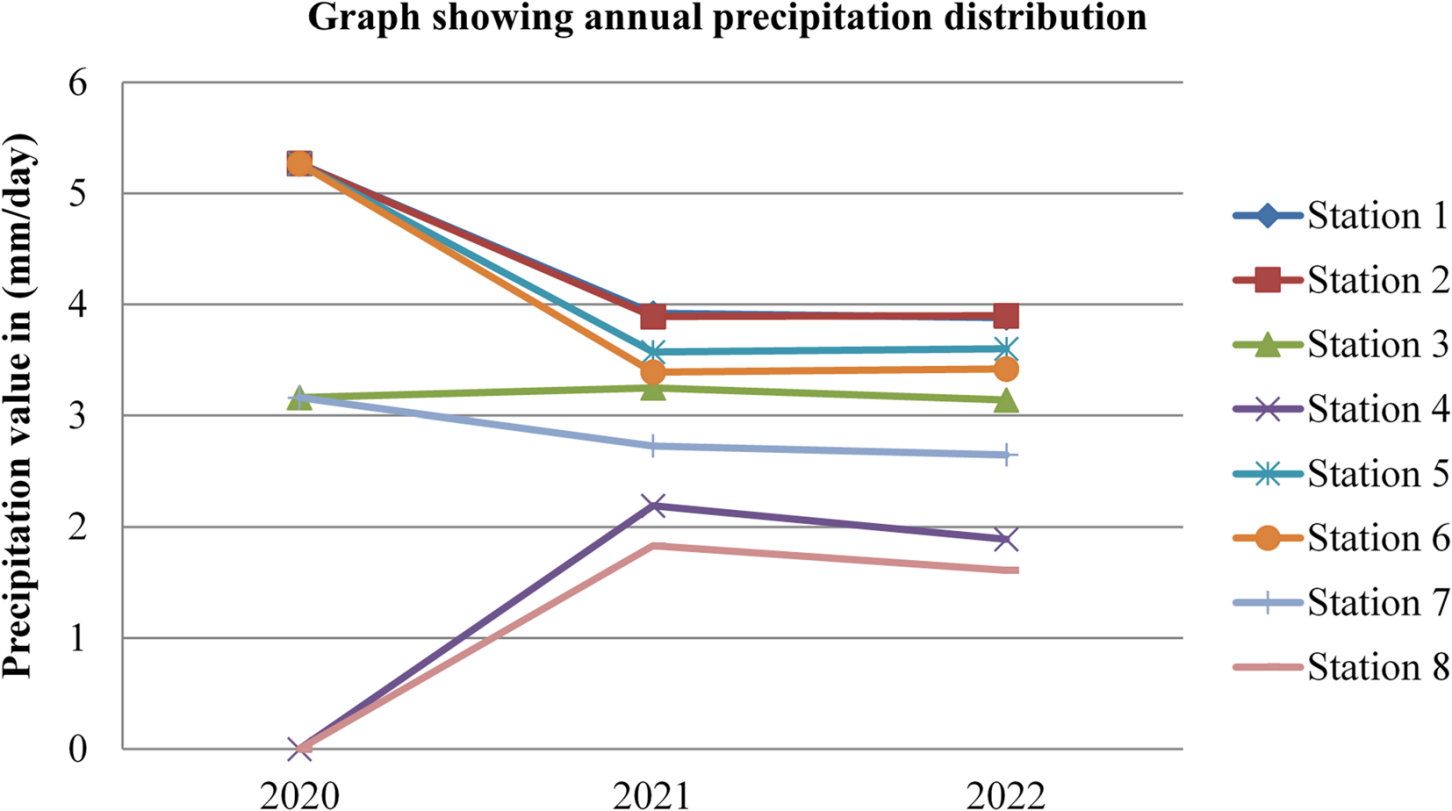
Annual precipitation of Dessie town from 2020–2022. Source^43^

Based on the annual precipitation data for Dessie town from the eight stations, here are the standard deviations and the corresponding error bars for each of the three years. The error bars represent the standard error of the mean (SEM), which is calculated as the standard deviation divided by the square root of the number of stations (*n* = 8).

**Table 4:** Precipitation statistical indicator of Dessie town from 2020–2022.

Statistical indicator	Years		
	2020	2021	2022
Mean	3.425	3.096	3.011
Standard deviation (SD)	2.306	0.775	0.880
Standard error of the mean (SEM)	0.815	0.274	0.311
Error bars (mean ± SEM)	3.425 ± 0.815	3.096 ± 0.274	3.011 ± 0.311

Source^43^.

From Fig. 18; Table 4, we understand that the annual variations of precipitation at each station for three consecutive years are almost similar, which implies that the annual concentrations of radon gas are constant or the variation in radon gas concentrations annually is almost small for each station and its statistical indicator result almost similar. This shows that there is a small annual variation in precipitation, which gives a small annual variation in radon gas. The precipitation variation is similar to radon gas concentration variations, and the result of the annual variation of radon gas is small. This result agree with previous results obtained like in 4 years of continuous monitoring in Milan, Italy conducted by^24^ variation of annual radon concentrations was up to 13% and a study conducted by^30^ found that the annual average outdoor radon concentration measured for a few consecutive years did not show significant ($\:<15\%$) differences between annual concentrations. Another finding, also conducted in Slovenia by^10^ and in Ethiopia by^33^, shows no significant difference between outdoor radon concentrations in any of the 4 seasons (annual).

### Ranges of radon-222 hosting nuclide (U-238)

The concentrations of uranium in basaltic rock type were shown in the given Table 1 below for our study area stations which hosting uranium element.

**Table 5:** Uranium concentrations in basaltic rocks.

Symbol	Type	Area ($\:{m}^{2}$)	Concentration in Ppm	Evaluated values in ppm
A1	Undifferentiated alluvial, elluvial, and lacustrine sediment	42009375.448	0.01–1	1
A2	Undifferentiated alluvial, elluvial, and lacustrine sediment	1708515.270	0.01–1	1
A3	Undifferentiated alluvial, elluvial, and lacustrine sediment	85719.486	0.01–1	1
A4	Undifferentiated alluvial, elluvial, and lacustrine sediment	213528.779	0.01–1	1
B1	Ashangi basalt	86835275.752	0.01–1	1
B2	Ashangi basalt	171352.780	0.01–1	1
C1	Dessie basalt	32686294.274	0.01–1	1
C2	Dessie basalt	2831830.148	0.01–1	1
C3	Dessie basalt	3403006.081	0.01–1	1
C4	Dessie basalt	1506100.747	0.01–1	1
C5	Dessie basalt	1094252.839	0.01–1	0.01
C6	Dessie basalt	820689.629	0.01–1	0.01
D	Main town basalt	13675154.295	0.01–1	1

Source^7^.

From Table 5 we had seen uranium concentrations estimated based on geological and geospatial factors (geological formation and climatic factors) for each type of basaltic rocks as we know from reports by^7^ basaltic rock contain uranium concentrations in the range of 0.01 to 1 ppm so, basaltic rocks denoted by letters A1.A2, A3, A4, B1, B2, C1, C2, C3, C4 and D have values 1 ppm greater uranium (radon gas) concentrations which implies that these stations have low soil moisture content, low relative humidity and low precipitations but high wind speed in summer season (Ethiopia’s Bega season). In contrast, rock types denoted by C5 and C6 have values 0.01ppm with low uranium (radon gas) concentrations, which implies that high soil moisture content, high relative humidity, and high precipitation, but low wind speed in winter (Ethiopia’s summer season).

### Calculation of radon concentrations in $\:Bq{m}^{-3}$ from uranium concentrations in Ppm

According to reports by^48^ ($\:1ppm\:of\:U-238=12.35\:{Bq\:kg}^{-1}$), based on this conversion, we calculated uranium activity concentrations in $\:{Bqm}^{-3}$ from concentrations expressed in ppm by using Eq. (3.1).

3.1 $$\begin{gathered} {\text{Activity concentrations of uranium in}}\,\,Bq{m^{ - 3}}\,\,{\text{in basalt rock}}\,\,({A_{U - 238}}in~Bq{m^{ - 3}})={\text{Activity concentrations of uranium in}}\,\,Bqk{g^{ - 1}} \hfill \\ *{\text{Density of basalt rock in}}\,\,kg{m^{ - 3}} \hfill \\ \end{gathered}$$

For 1 ppm of U-238, we calculated activity concentrations as follows:

The density of basalt rock is an average value of 2900$\:\:kg{m}^{-3}$^49^

$$\:{A}_{U-238}in\:{Bqm}^{-3}=12.35\:{Bq\:kg}^{-1}*\:2900\:kg{m}^{-3}=\text{35,815}\:\:{Bqm}^{-3}$$

To estimate radon activity concentrations from 1 ppm of U-238, we used Eq. (3.2).

3.2 $${\text{Radon activity concentrations in}}\,Bq{m^{ - 3}}={\text{Uranium activity concentrations in}}\,\,Bq{m^{ - 3}}\,\,*\,\,{\text{E}}$$

$\:{A}_{Rn-222}\:in\:{Bqm}^{-3}={A}_{U-238}in\:{Bqm}^{-3}*E$, where E is the radon gas emanation coefficient, which has a value of 0.1 to 0.3 for basalt rock, as IAEA reports.

For an emanation coefficient (E) = 0.1 (10%)

$$\:{A}_{Rn-222}\:in\:{Bqm}^{-3}={A}_{U-238}in\:{Bqm}^{-3}*E=\text{35,815}{\:Bqm}^{-3}*0.1=\text{3,581.5}\:{\:Bqm}^{-3}$$

For an emanation coefficient (E) = 0.3 (30%)

$$\:{A}_{Rn-222}\:in\:{Bqm}^{-3}={A}_{U-238}in\:{Bqm}^{-3}*E=\text{35,815}{\:Bqm}^{-3}*0.3=\text{10,744.5}\:{\:Bqm}^{-3}$$

Depending on this emanation coefficient, the estimated radon activity concentrations from $\:\text{3,5815}\:{\:Bqm}^{-3}$ of uranium could range from$\:\text{3,581.5}\:{\:Bqm}^{-3}\:to\:\text{10,744.5}\:{\:Bqm}^{-3}\:$for 1 ppm of uranium concentration in basaltic rocks.

To get uranium activity concentrations in $\:\varvec{B}\varvec{q}{\varvec{m}}^{-3}$ from uranium concentrations expressed in ppm by using Eq. (3.1).

For 0.01 ppm of uranium-238, we got the following result.

$$\:0.01ppm\:of\:U-238=0.1235\:{Bq\:kg}^{-1}$$

$$\:{\:\:\:A}_{U-238}in\:{Bqm}^{-3}=0.1235\:{Bq\:kg}^{-1}*\:2900\:kg{m}^{-3}$$

$$\:{A}_{U-238}in\:{Bqm}^{-3}=358.15\:{\:Bqm}^{-3}\:\:$$

Similarly, to find radon activity concentrations from 0.01 ppm of U-238 activity concentrations, we used Eq. (3.2).

For an emanation coefficient (E) = 0.1 (10%)

$$\:{A}_{Rn-222}\:in\:{Bqm}^{-3}={A}_{U-238}in\:{Bqm}^{-3}*E=358.15\:{\:Bqm}^{-3}\:*0.1=35.815{Bqm}^{-3}\:$$

For an emanation coefficient (E) = 0.3 (30%)

$$\:{A}_{Rn-222}\:in\:{Bqm}^{-3}={A}_{U-238}in\:{Bqm}^{-3}*E=358.15\:{\:Bqm}^{-3}*0.3=107.445\:{Bqm}^{-3}$$

Depending on this emanation coefficient, the estimated radon activity concentrations from $\:358.15\:{\:Bqm}^{-3}$ of uranium could range from$\:35.815\:{\:Bqm}^{-3}\:to\:107.445\:{\:Bqm}^{-3}\:$.

**Table 6:** Radon activity concentrations in $\:\text{B}\text{q}{\text{m}}^{-3}$ from uranium concentrations.

Symbol	Type of rocks	Volume ($\:{m}^{3}$)	Evaluated values of uranium concentrations in ppm	Activity of radon concentrations in $\:Bq{m}^{-3}$
A1	Undifferentiated alluvial, elluvial, and lacustrine sediment	42009375.448	1	$\:\text{3,581.5}-\text{10,744.5}\:$
A2	Undifferentiated alluvial, elluvial, and lacustrine sediment	1708515.270	1	$\:\text{3,581.5}-\text{10,744.5}$
A3	Undifferentiated alluvial, elluvial, and lacustrine sediment	85719.486	1	$\:\text{3,581.5}-\text{10,744.5}$
A4	Undifferentiated alluvial, elluvial, and lacustrine sediment	213528.779	1	$\:\text{3,581.5}-\text{10,744.5}$
B1	Ashangi basalt	86835275.752	1	$\:\text{3,581.5}-\text{10,744.5}$
B2	Ashangi basalt	171352.780	1	$\:\text{3,581.5}-\text{10,744.5}$
C1	Dessie basalt	32686294.274	1	$\:\text{3,581.5}-\text{10,744.5}$
C2	Dessie basalt	2831830.148	1	$\:\text{3,581.5}-\text{10,744.5}$
C3	Dessie basalt	3403006.081	1	$\:\text{3,581.5}-\text{10,744.5}$
C4	Dessie basalt	1506100.747	1	$\:\text{3,581.5}-\text{10,744.5}$
C5	Dessie basalt	1094252.839	0.01	$\:35.815-107.445\:\:$
C6	Dessie basalt	820689.629	0.01	$\:35.815-107.445$
D	Main town basalt	13675154.295	1	$\:\text{3,581.5}-\text{10,744.5}$

Source (QGIS 3.34.0).

From Table 6, we understand that the radon gas activity concentrations have values in the range$\:(\text{3,581.5}-\text{10,744.5})\:\:Bq{m}^{-3}$ for positions labeled by A1, A2, A3, A4, B1, B2, C1, C2, C3, C4 and D and $\:(35.815-107.445)$ $\:Bq{m}^{-3}$for positions labeled by C5 and C6. These findings indicate that the estimated radon activity concentration for 1ppm of U-238 range of approximately$\:\:\text{3,581.5}\:Bq{m}^{-3}\:to\:\text{10,744.5}\:Bq{m}^{-3}$, derived from a uranium activity concentration of $\:\text{35,815}\:\:{Bqm}^{-3}$ in basalt rock, provides some insights regarding environmental health. Radon concentrations in this range are significantly higher than typical outdoor levels, which are generally between$\:\:1\:and\:100\:Bq{m}^{-3}$, with an estimated annual average of around $\:10\:Bq{m}^{-3}$ as highlighted by^9^.

The World Health Organization (WHO) has set an average concentration reference level of 100 $\:Bq{m}^{-3}$for indoor radon, but there is no specific reference level for outdoor radon concentrations. Outdoor radon concentrations are generally much lower than indoor levels, often by an order of magnitude or more. Typical outdoor radon ranges between 1 and 100 $\:Bq{m}^{-3}$, with an estimated annual average of around 10 $\:Bq{m}^{-3}$ according to^9^. While there is no single “world accepted value” for outdoor radon, typical levels are in the range of 1-100$\:Bq{m}^{-3}$, with an average around 10$\:Bq{m}^{-3}$, according to the limited data available in the literature. Outdoor radon is an important consideration for radiation protection, even though indoor radon poses a greater health concern. For 0.01 ppm of U-238, the finding of activity concentrations of Rn-222 at positions labeled by C5 and C6 indicates that it agrees with the previous findings value between$\:\:1\:and\:100\:Bq{m}^{-3}$^9^.

### Mapping of radon gas distributions

**Fig. 19:**
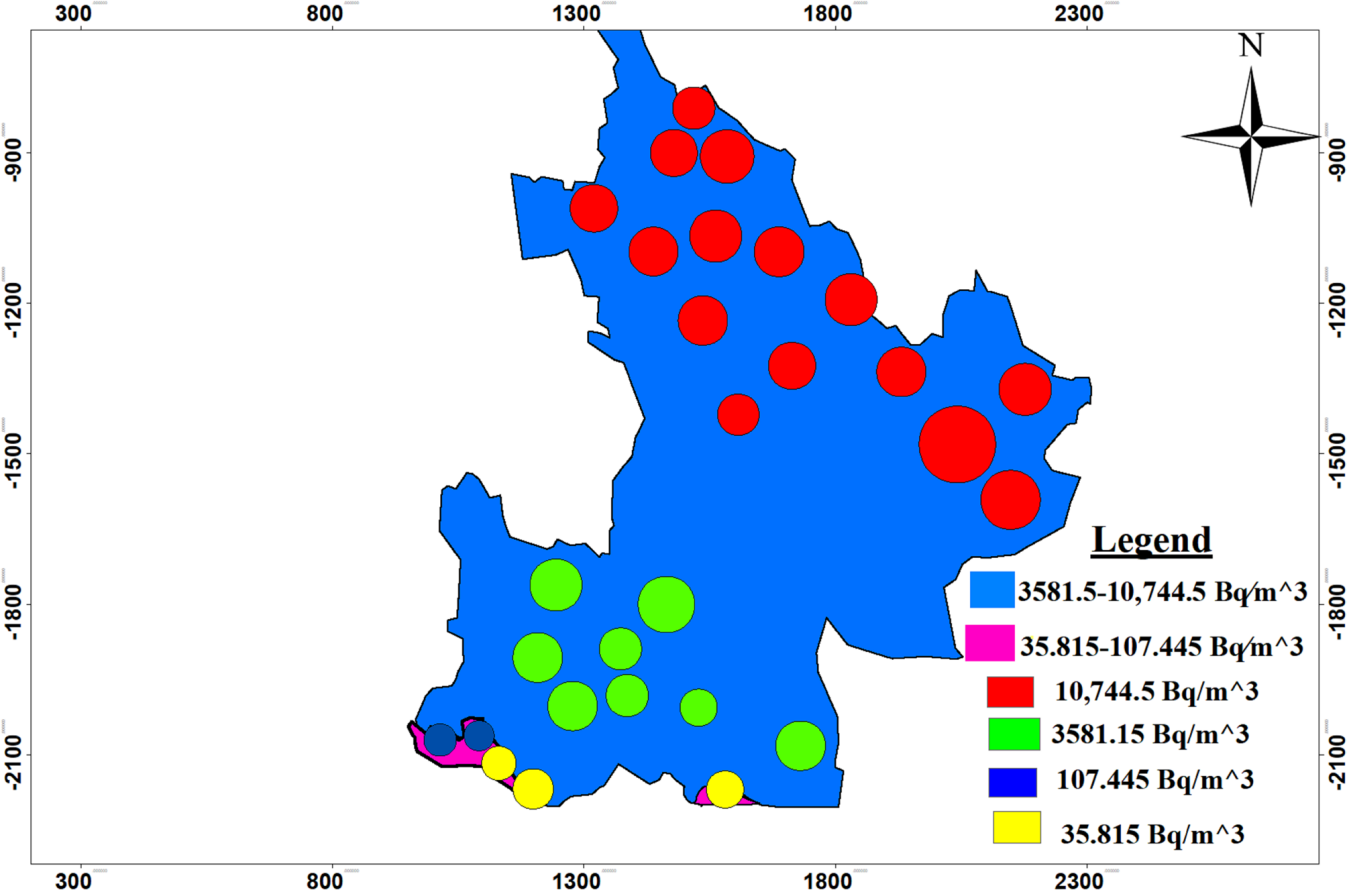
Radon gas distribution map.

The radon gas distribution map shown below in Fig. 19 highlights radon gas activity concentrations in Dessie town based on geological and geospatial data analysis results. The finding indicates that radon gas activity concentrations in the range between $\:\text{3,581.5}-\text{10,744.5}\:\:Bq{m}^{-3}$ values painted in blue color have elevated radon gas activity concentrations at stations labeled by A1, A2, A3, A4, B1, B2, C1, C2, C3, C4 and D. Based on this radon gas activity concentrations values some places have approximate value to $\:\text{10,744.5}\:\:Bq{m}^{-3}$as we circled red on the map shown below similarly places which have value$\:\text{3,581.5}\:Bq{m}^{-3}$ circled green have relatively small radon gas activity concentration as compared with red circled. Whereas radon gas activity concentrations in the range between $\:35.815-107.445\:\:\:Bq{m}^{-3}$ values painted in pink color at stations C5 and C6 have smaller radon gas activity concentrations values relative to other station points, but radon gas activity concentration values at these points are within the accepted value ($\:1\:and\:100\:Bq{m}^{-3}$).

### Implications for environmental health

We know that radon gas is a leading cause of lung cancer, next to smoking, and is a known carcinogen. So its impact on environmental health depend on its activity concentrations, but in our study area we had verified that elevated radon gas activity concentrations at stations labeled by A1, A2, A3, A4, B1, B2, C1, C2, C3, C4 and D low radon gas activity concentrations at stations labeled by C5 and C6 as we had shown in the above result table and maps. This implies that there exists health related problem with radon gas radiation in Dessie town, especially at stations A1, A2, A3, A4, B1, B2, C1, C2, C3, C4 and D. The presence of elevated radon concentrations throughout Dessie town implies a widespread public health concern. As radon is a known human carcinogen, especially for lung cancer, prolonged exposure at all stations, even those with lower concentrations, poses a health risk. This finding emphasizes the need for public awareness and potential mitigation strategies to protect the community from long-term exposure.

## Conclusion and recommendation

### Conclusion

The geological foundation of Dessie town, characterized by basaltic rock formations, has a direct impact on the distribution of radon gas. The study’s measurements show a clear variation in radon activity concentrations across different locations, highlighting specific areas of concern. Radon gas activity concentrations in different stations have values in the range between $\:\text{3,581.5}-\text{10,744.5}\:\:Bq{m}^{-3}$ for stations labeled by A1, A2, A3, A4, B1, B2, C1, C2, C3, C4 and D, and for stations labeled by C5 and C6 have radon gas activity concentration values have in the range between$\:\:35.815-107.445\:\:\:Bq{m}^{-3}$. Based on this result, there is a potential hotspot place, especially painted red, circled on the map for radon gas activity concentrations values between $\:\text{3,581.5}-\text{10,744.5}\:\:Bq{m}^{-3}\:$and this value is above the typical outdoor radon gas activity concentration ranges between 1 and 100 $\:Bq{m}^{-3}$, with an estimated annual average of around 10 $\:Bq{m}^{-3}$ according to^9^. But at stations C5 and C6, radon gas activity concentration values are in the range between $\:35.815-107.445\:\:Bq{m}^{-3}$ and these also have small stations that have a little bit of hot spot places as circled with blue color, and this value is almost in the range of accepted typical outdoor radon gas activity concentration values. This implies that there exists health related problem with radon gas exposure in Dessie town for prolonged exposure at all stations, even if the degree of health impact varies. This study serves as a critical first step in an environmental impact assessment for Dessie town, providing essential data for urban planners and public health officials.

### Recommendation

The results highlight the importance of radon monitoring, especially in areas with high natural radioactivity or uranium-bearing geological formations like basalt. Outdoor radon measurements can serve as a baseline to assess anthropogenic contributions to radon exposure and identify potential radon priority areas.

The calculated radon levels, based on the uranium activity in basalt rock, suggest the potential for high radon exhalation rates from this type of geological formation. However, actual radon measurements would be needed to confirm these estimates and assess the potential health impact. Based on the result of our finding that Dessie town is surrounded by basalt rock and its radon gas concentration values are in two ranges from $\:\text{3,581.5}t\:o\text{10,744.5}\:\:Bq{m}^{-3}$ and$\:\:35.815-107.445\:\:\:Bq{m}^{-3}$. Finally, we recommend that for future researcher who wants to study such an issue, it is preferable to measure radon gas activity concentrations experimentally by taking samples at each station from the study area’s geological formations in order to get more accurate results.

## Supplementary Information

Below is the link to the electronic supplementary material.

Supplementary Material 1

## Data Availability

The climate factor data sets generated and analyzed during the current study for the study area are available in https://power.larc.nasa.gov/data-access-viewer and the geological map data for the study area was adopted from the Ethiopian Geological Institute as presented in appendix 2.
