# Supplementary figures and images for: Radon gas mapping for environmental assessment in Dessie, Ethiopia
Source: Sci Rep. 2025 Oct 24;15:37271. doi: 10.1038/s41598-025-21201-4 (PMC12552722; doi:10.1038/s41598-025-21201-4)

**APPENDIX- 1**


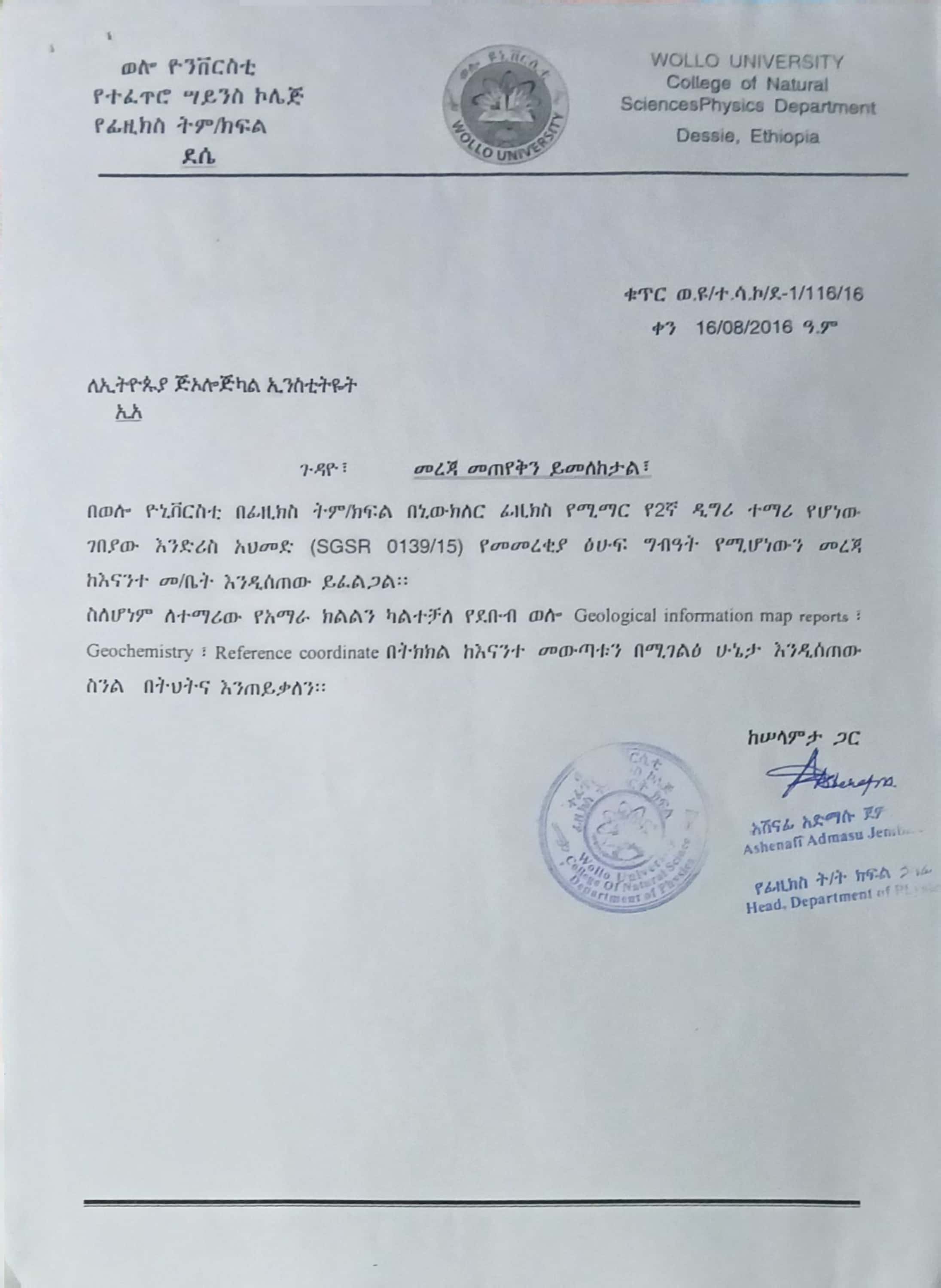


APPENDIX- 2

Dessie geological map obtained from Ethiopian Geological Institute

Supplement: Supplementary file 1 — Supplementary Material 1 [file 41598_2025_21201_MOESM1_ESM.docx]
